# Supplementary material for: Profiling variable-number tandem repeat variation across populations using repeat-pangenome graphs
Source: Nat Commun. 2021 Jul 12;12:4250. doi: 10.1038/s41467-021-24378-0 (PMC8275641; doi:10.1038/s41467-021-24378-0)
Supplement: Supplementary file 1 — Supplementary information [file 41467_2021_24378_MOESM1_ESM.pdf]

Supplementary information

Table of contents

Supplementary Figures **3**

Supplementary Figure 1. Comparison between the tandem repeat database in GangSTR and this work. **3**

Supplementary Figure 2. An example of multiple STR annotations within a VNTR. **4**

Supplementary Figure 3. An example VNTR annotation split by adVNTR-NN. **5**

Supplementary Figure 4. Completeness of VNTR annotations in individual genomes. **6**

Supplementary Figure 5. Classes of VNTRs removed by alignment quality filtering. **7**

Supplementary Figure 6. LSB at repetitive regions. **8**

Supplementary Figure 7. LSB at non-repetitive regions of all genotyped samples. **9**

Supplementary Figure 8. LSB at non-repetitive regions preserves the relation between samples at repetitive regions. **10**

Supplementary Figure 9. Nearest neighbor search for LSB at VNTR regions using LSB at nonrepetitive regions as a proxy. **11**

Supplementary Figure 10. Profile of prediction accuracy for each sample. **12**

Supplementary Figure 11. Performance of per-locus length prediction accuracy relative to GRCh38. **13**

Supplementary Figure 12. Correlation between the estimation error in VNTR length and in LSB. **13**

Supplementary Figure 13. Example of deviation in LSB across samples. **14**

Supplementary Figure 14. Distribution of length estimation error for loci with or without a missing haplotype. **14**

Supplementary Figure 15. Correlation between length estimation error and fraction of novel k-mers. **15**

Supplementary Figure 16. Relationship between GC content and length prediction error. **15**

Supplementary Figure 17. Effect of GC content change on bias and length estimation. **16**

Supplementary Figure 18. Examples of unstable loci with individuals > 10 standard deviations above the mean. **16**

Supplementary Figure 19. Null and observed distributions of kmcd and rd2 between the EAS and AFR populations. **17**

Supplementary Figure 20. Distance of TRs and eTRs to telomere. **17**

Supplementary Figure 21. Association between the top 50 pairs of eVNTR and eGene. **18**

Supplementary Figure 22. Conditional association of chr5:96896863-96896963 VNTR with ERAP2 expression over chr5_96916885_T_C_b38. **23**

Supplementary Figure 23. Linkage disequilibrium (LD) between chr5:96896863-96896963 VNTR and nearby SNPs. **23**

Supplementary Figure 24. Spurious alignment of Illumina reads to GRCh38 at a VNTR locus. **24**

Supplementary Figure 25. Boundary expansion recovers the proper boundary of VNTR alleles. **25**

Supplementary Figure 26. Distribution of number of genes overlapping shuffled high VST loci. **26**

Supplementary Figure 27. Distribution of genes and UTR regions overlapping shuffled unstable loci. **26**

Supplementary Figure 28. Number of eVNTRs shared between or specific to each tissue. **27**

Supplementary Figure 29. Length distribution of VNTRs and eVNTRs. **28**

Supplementary Figure 30. Sample QC on VNTR genotypes of the 1000 Genomes. **29**

Supplementary Figure 31. Sample QC on VNTR genotypes the GTEx Genomes. **30**

Supplementary Figure 32. Growth of relative VNTR-graph size. **31**

Supplementary Figure 33. Example of under-alignment of orthologous VNTR sequences by pggb. **31**

Supplementary Figure 34. Misalignment of simulated VNTR reads by bwa. **32**

Supplementary Figure 35. Misalignment of VNTR reads to GRCh38 rescued by danbing-tk. **32**

Supplementary Figure 36. Relationship between VNTR length and prediction error. **33**

Supplementary Figure 37. Relationship between eVNTR P-value and prediction error. **33**

Supplementary Figure 38. Comparing the alignment accuracy with and without threading. **34**

Supplementary Figure 39. Replication of Vst on the 698 genomes related to the 1KGP samples. **35**

Supplementary Figure 40. Incremental RPGG construction and change in boundary annotations. **36**

Supplementary Tables **37**

Supplementary Table 1. Initial VNTR discoveries **37**

Supplementary Table 2. False mapping of reads by danbing-tk over the initial 73,582 loci. **37**

Supplementary Table 3. eVNTRs discovered in this work that overlap with other studies **38**

Supplementary Table 4. Data source **39**

Supplementary Table 5. Augmenting database with disease-related tandem repeats **40**

Supplementary Table 6. Comparison of alignment statistics between danbing-tk and GraphAligner. **40**

Supplementary Table 7. Realignment statistics of misaligned VNTR reads from bwa. **41**

Supplementary Notes **42**

Supplementary Note 1. The full list of HGSVC members. **42**

Supplementary Figures

Supplementary Figure 1. Comparison between the tandem repeat database in GangSTR and this work.

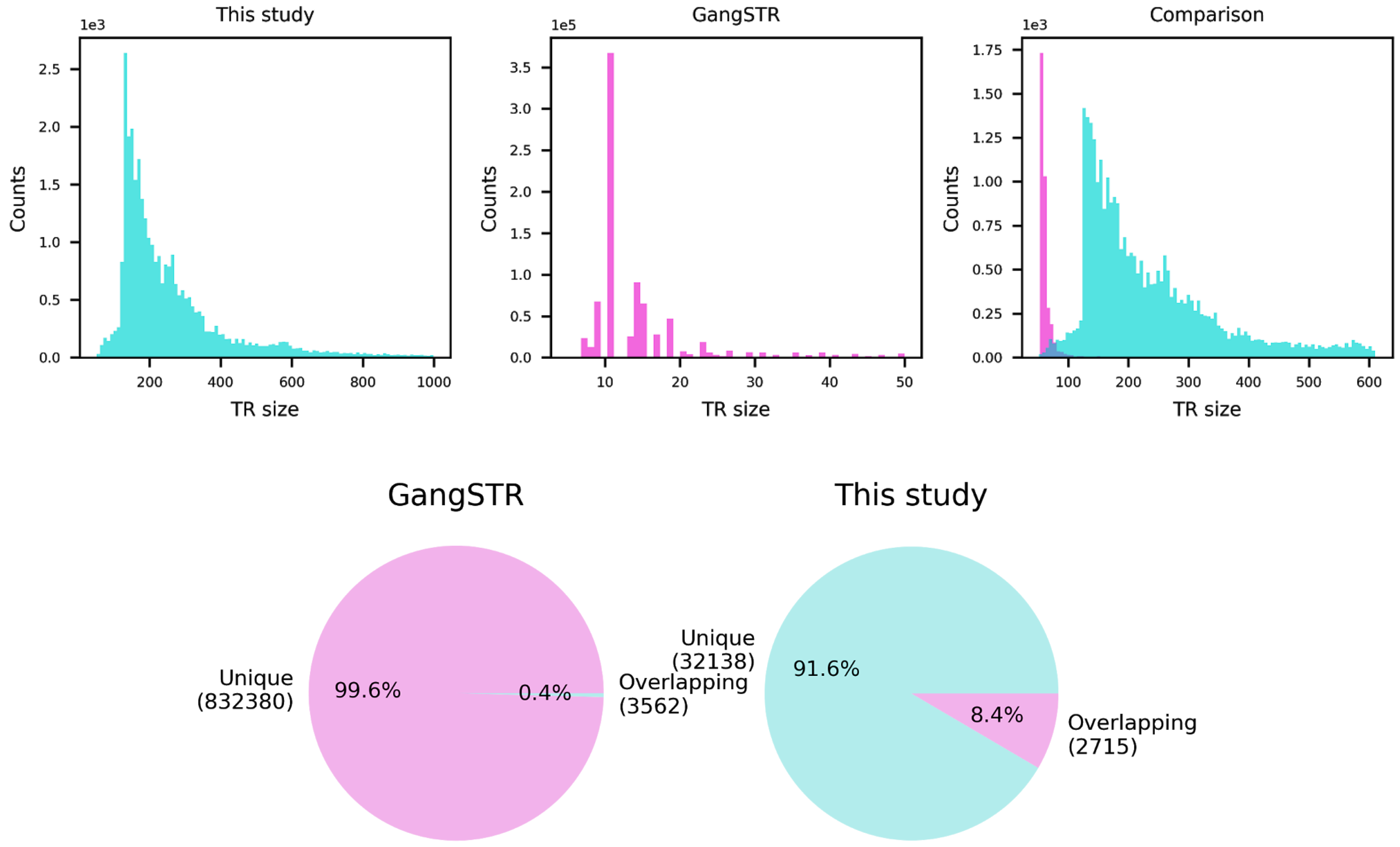

a, Size distribution of the TRs annotated in each study. TRs with size greater than 150 bp in at least one assembly and with size greater than 50 bp in hg38 are annotated in this study. Tandem repeat sizes above 1000 bp, above 50 bp, and below 50 bp are not shown for this study (left), GangSTR (middle) and comparison (right), respectively. **b,** Percentage of overlapping TRs between databases. The number of overlapping loci changes across databases since multiple loci in GangSTR's database could correspond to only one locus in our database. Source data are provided as a Source Data file.

Supplementary Figure 2. An example of multiple STR annotations within a VNTR.

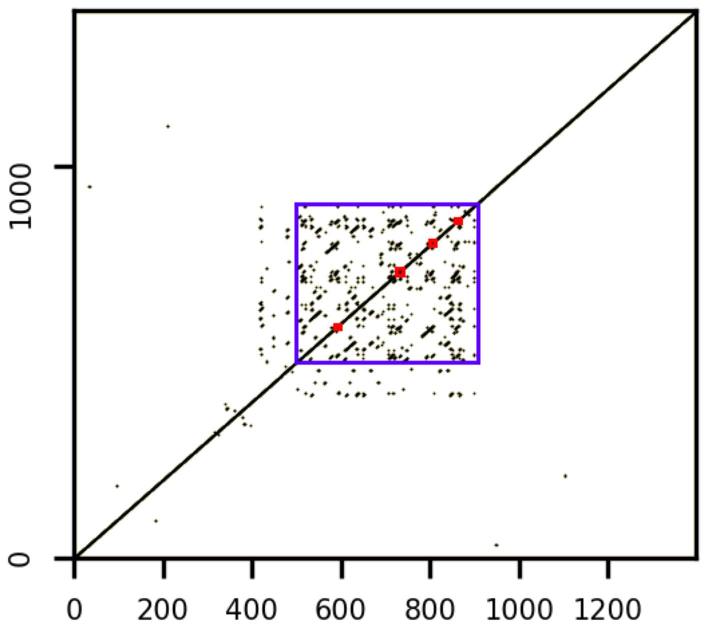

Dot plot was generated using exact matching between 9-mers along chr1:861277-862683. Annotations of four STRs (red box; chr1:861863-861874, chr1:862001-862016, chr1:862077-862088 and chr1:862133-862144) and one VNTR (blue box; chr1:861777-862183; before boundary expansion) are highlighted.

Supplementary Figure 3. An example VNTR annotation split by adVNTR-NN.

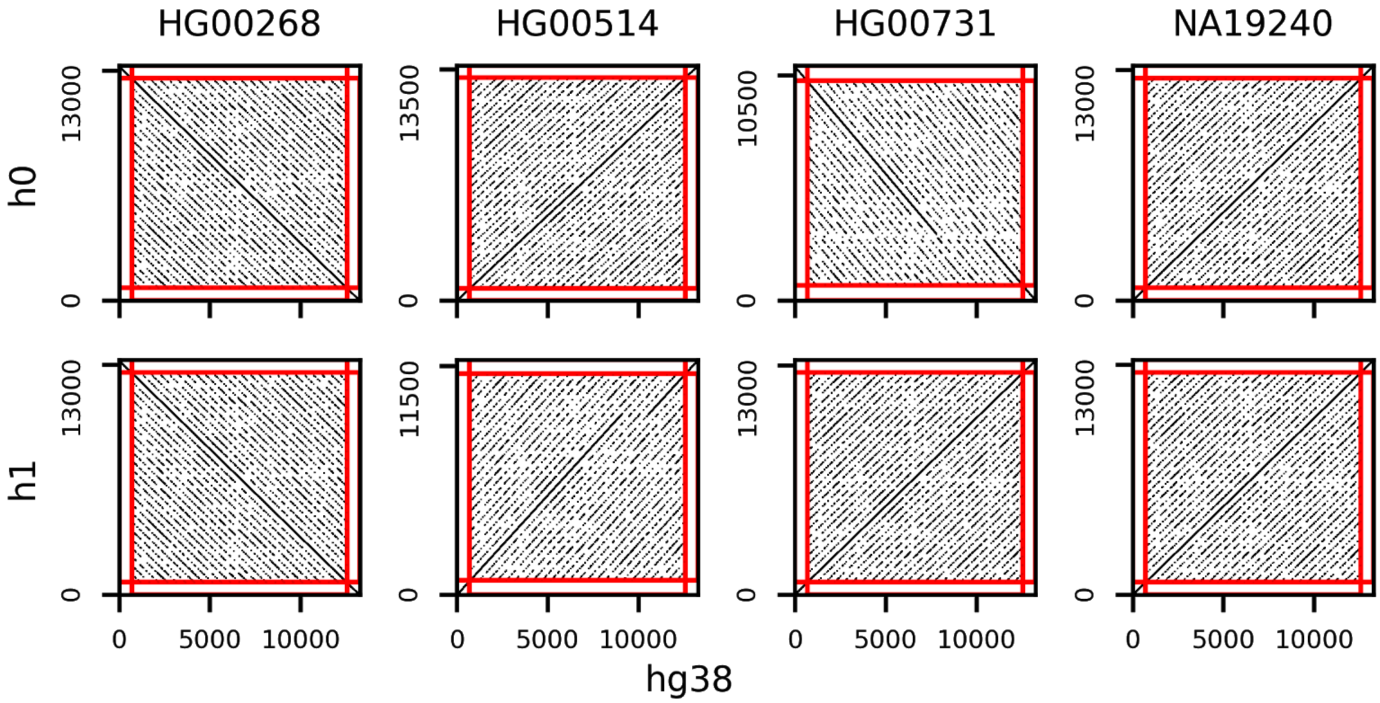

Dot plots of VNTR sequences at chr14:104941587-104953440 from four assemblies and GRCh38 are shown. Note that this region is split into 39 sub-regions in adVNTR-NN with an average VNTR size of 54 bp.

Supplementary Figure 4. Completeness of VNTR annotations in individual genomes.

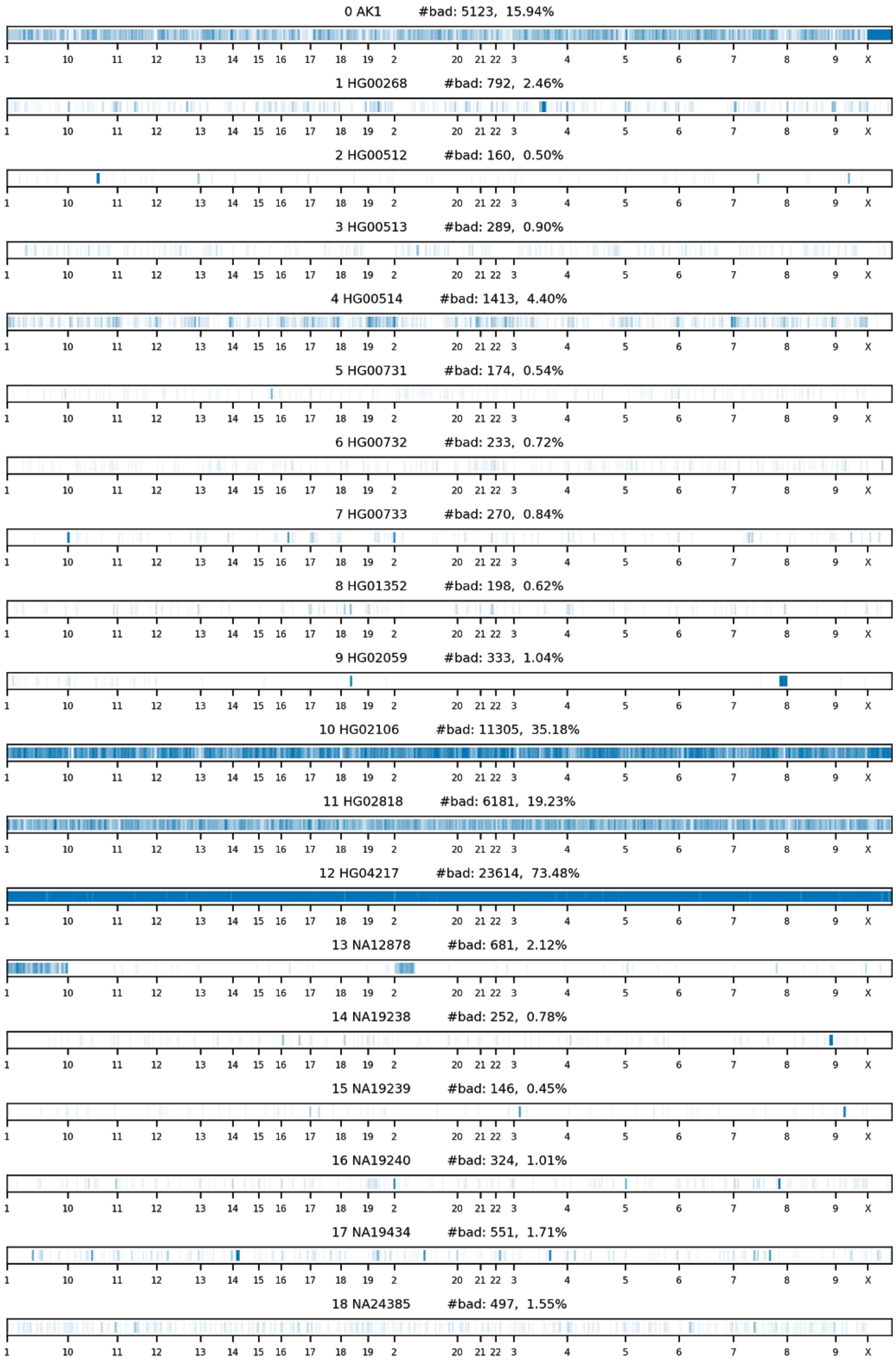

X-axis indicates relative genomic order of each missing VNTR locus which is marked by a blue stripe. A locus is called missing if both VNTR haplotypes in the genome are missing. Percentage of missing loci is the number of missing loci divided by 32,138, the total number of loci annotated. Source data are provided in Supplementary Data 2.

Supplementary Figure 5. Classes of VNTRs removed by alignment quality filtering.

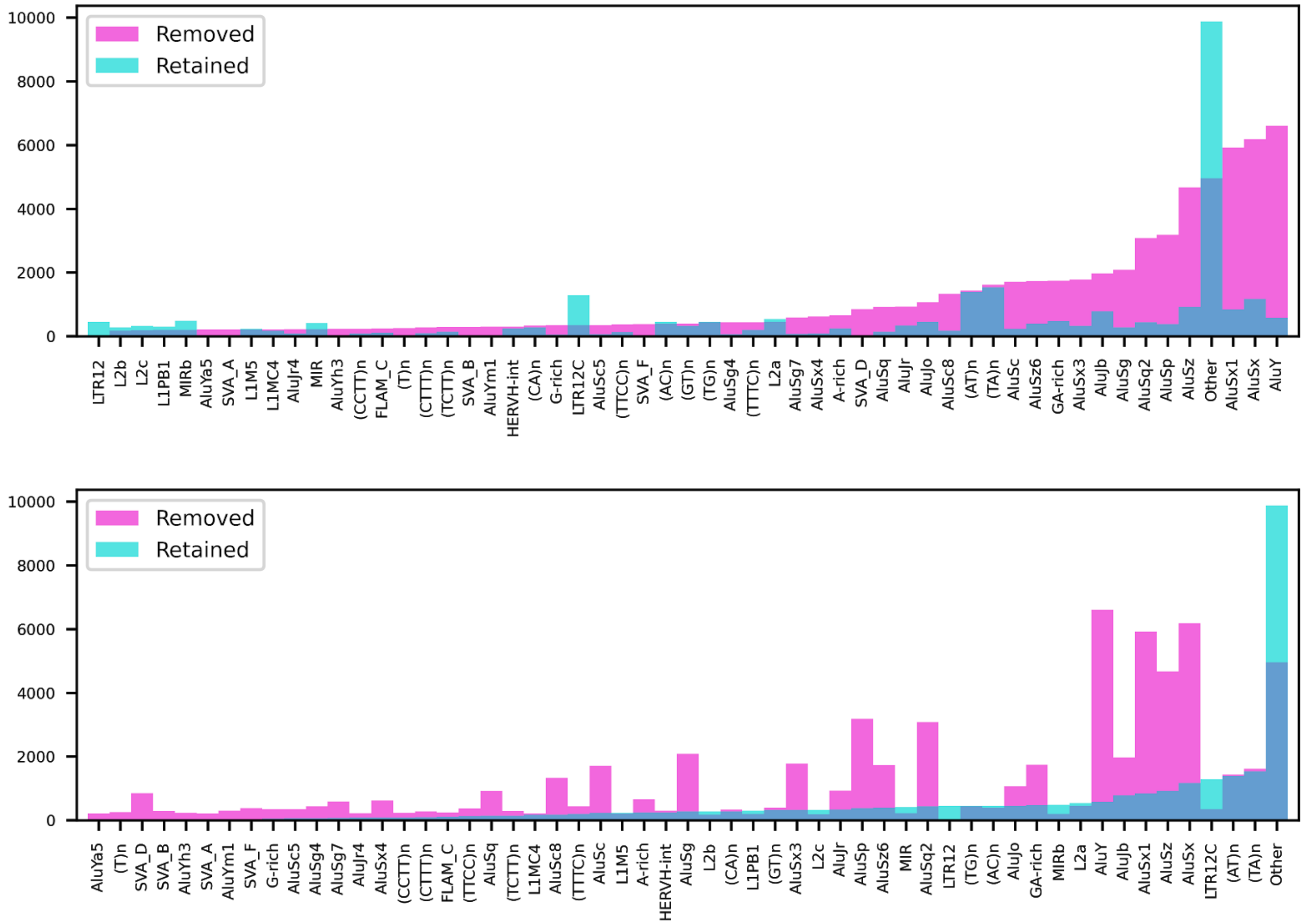

Annotations of VNTR classes are retrieved from the RepeatMasker track in UCSC Genome Browser. VNTRs that span multiple repeat annotations will be counted once for each class. Repeat classes are shown only for those with at least 200 repeats called. Class "other" indicates repeats not annotated in the RepeatMasker track. Labels on x-axis are sorted by the number of removed (top) or retained (bottom) loci. Source data are provided as a Source Data file.

Supplementary Figure 6. LSB at repetitive regions.

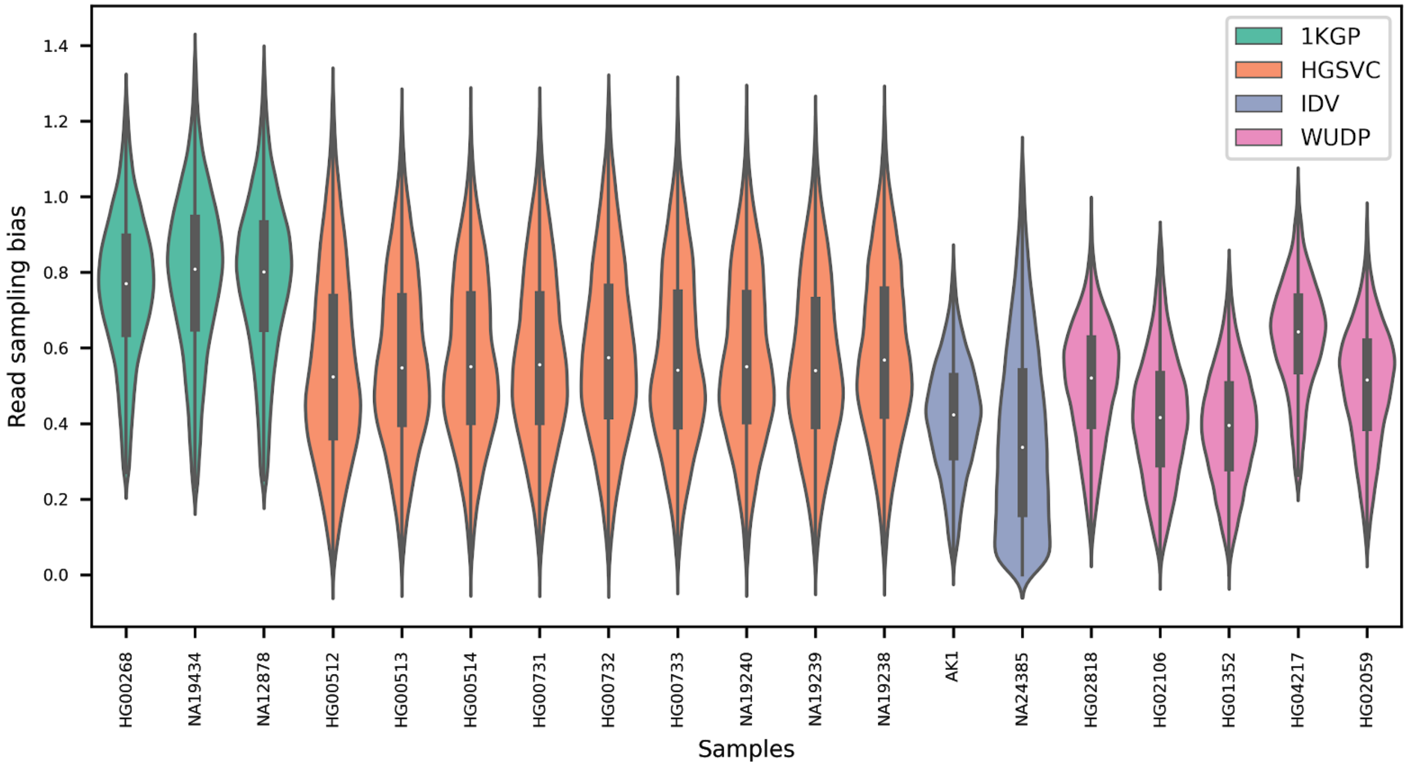

The distribution of biases (n=32,138) at the 32,138 genotyped loci are shown for each sample. The box within each density estimate spans from the lower quartile to the upper quartile, with the white dot indicating the median. Whiskers extend to points that are within 1.5 interquartile range (IQR) from the upper or the lower quartiles. Samples are retrieved from HGSVC, Human Genome Structural Variation Consortium datasets; 1KGP, 1000 Genomes Project datasets; WUDP, Washington University Diversity Project datasets; and IDV, individual studies. Source data are provided as a Source Data file.

Supplementary Figure 7. LSB at non-repetitive regions of all genotyped samples.

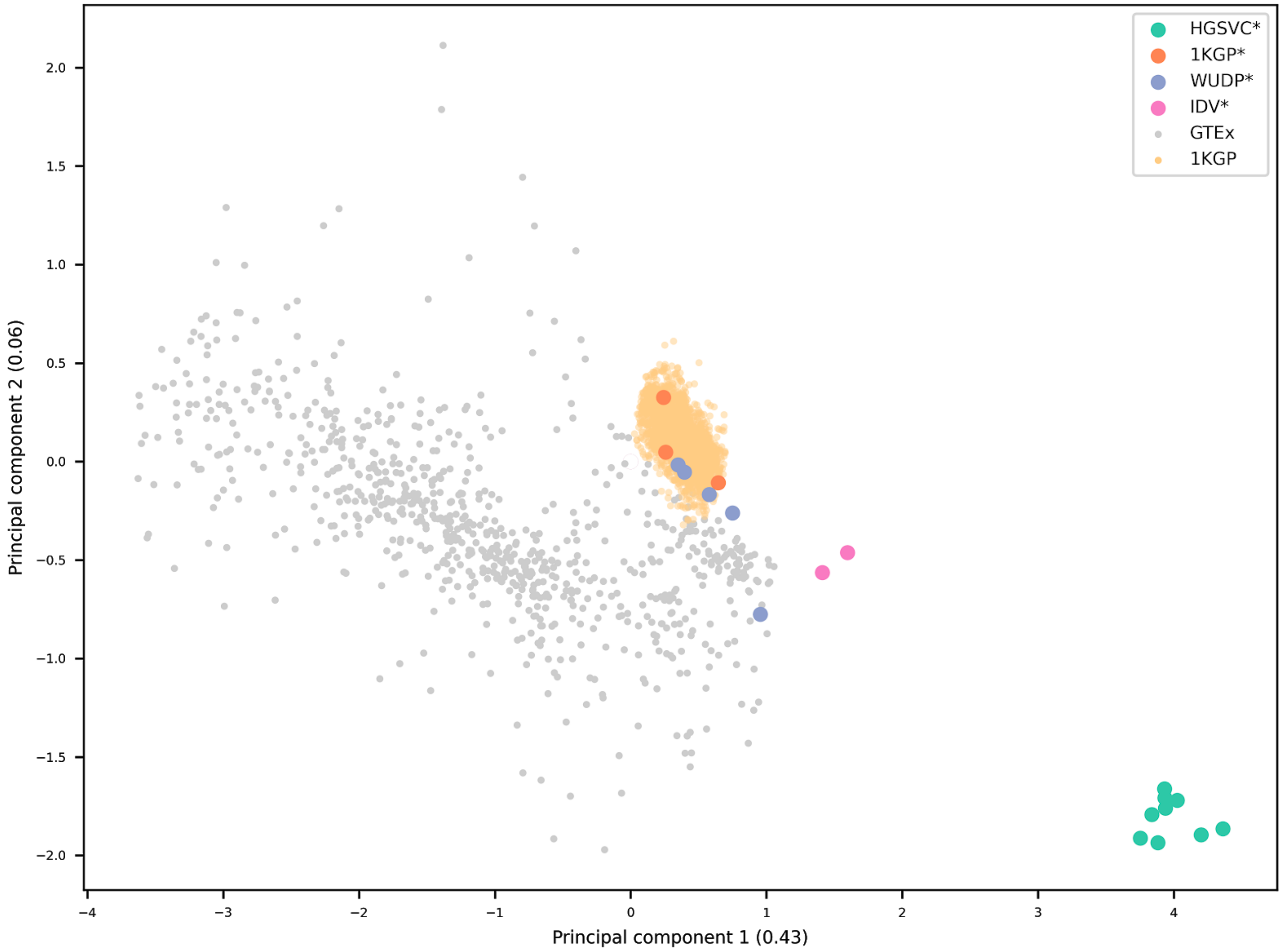

Principal component analysis was done on a $N \times L$ matrix, where N is the number of samples, and L is the number of unique regions. Each row of the matrix is a vector of LSB in 397 unique regions from a single sample. Each sample is a tuple of (genome, sequencing run). Samples are retrieved from HGSVC, Human Genome Structural Variation Consortium datasets; 1KGP, 1000 Genomes Project datasets; WUDP, Washington University Diversity Project datasets; and IDV, individual studies; asterisks indicate samples with haplotype-resolved assemblies available. Source data are provided as a Source Data file.

Supplementary Figure 8. LSB at non-repetitive regions preserves the relation between samples at repetitive regions.

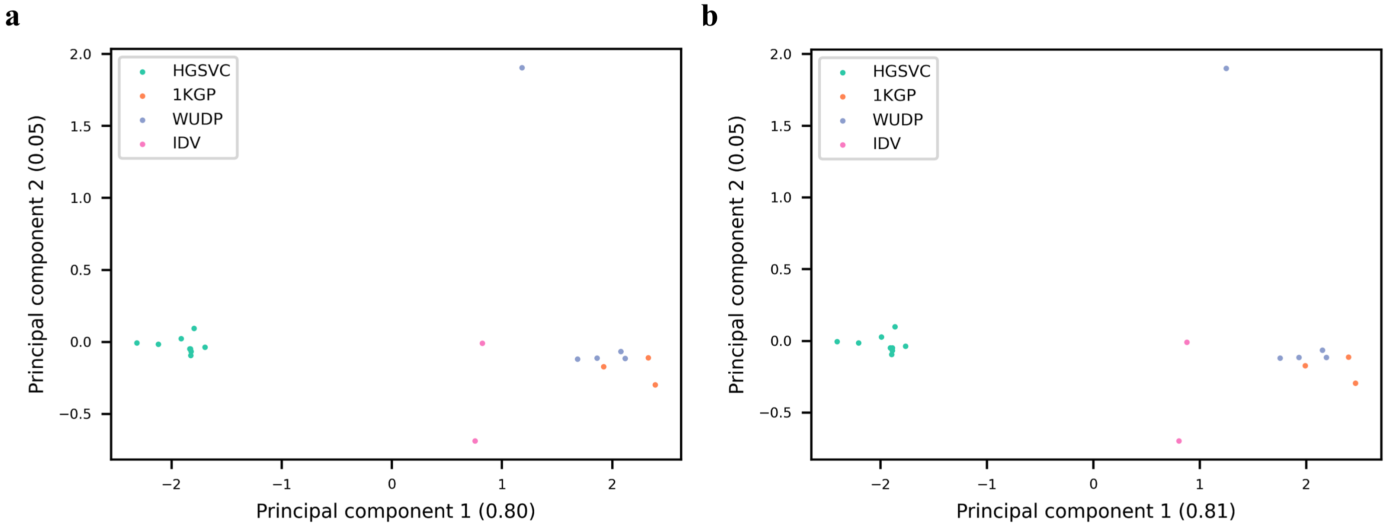

Principal component analysis of LSB in VNTR (**a**) and unique (**b**) regions. PCA was done on an $N \times L$ matrix, where N is the number of samples, and L is the number of VNTR loci. Each row of the matrix is a vector of LSBs in 32,138 VNTR regions from a single sample. Each sample is a tuple of (genome, sequencing run). Samples are retrieved from HGSVC, Human Genome Structural Variation Consortium datasets; 1KGP, 1000 Genomes Project datasets; WUDP, Washington University Diversity Project datasets; and IDV, individual studies. Source data are provided as a Source Data file.

Supplementary Figure 9. Nearest neighbor search for LSB at VNTR regions using LSB at nonrepetitive regions as a proxy.

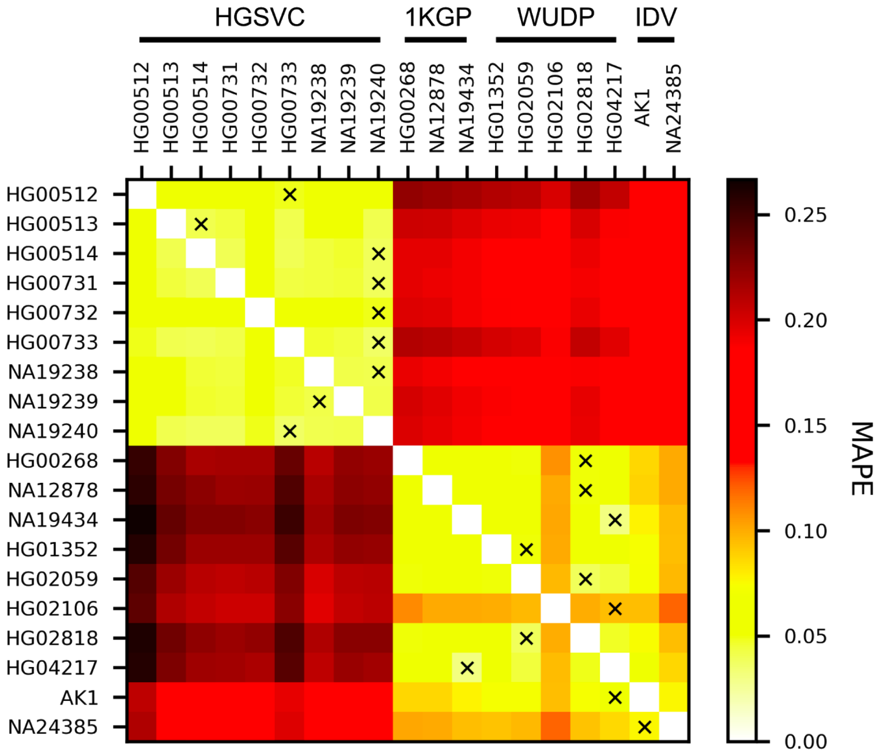

The heat map shows the mean absolute error between each pair of LSB at VNTR regions. For the sample denoted in each column, each cross indicates the nearest neighbor for that sample based on the LSB in nonrepetitive regions. HGSVC, Human Genome Structural Variation Consortium datasets; 1KGP, 1000 Genomes Project datasets; WUDP, Washington University Diversity Project datasets; IDV, individual studies. Source data are provided as a Source Data file.

Supplementary Figure 10. Profile of prediction accuracy for each sample.

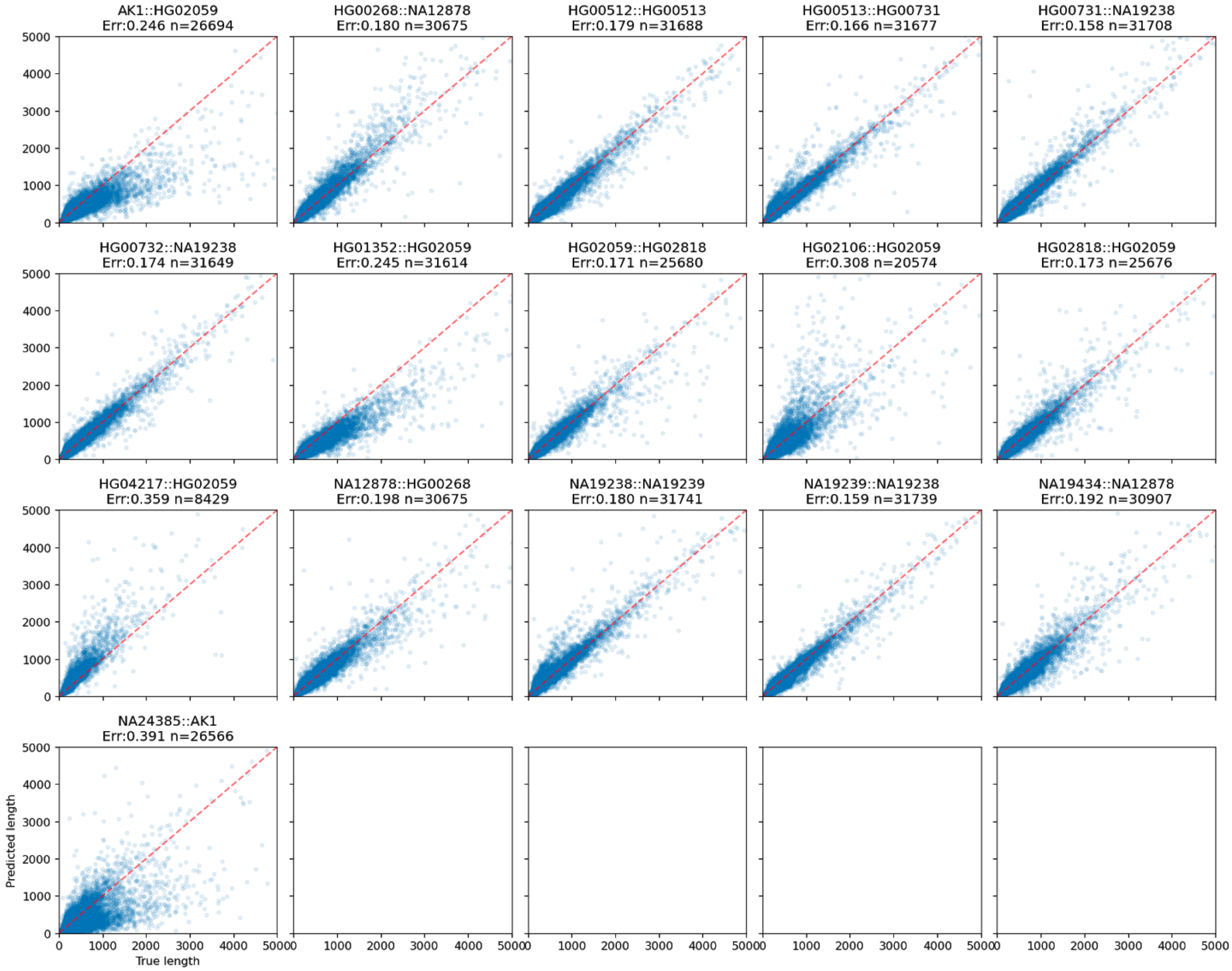

True and predicted lengths are plotted against each other for each sample. Each subtitle shows the sample name followed by its nearest sample, mean absolute percentage error and the number of loci. Loci not annotated in either the sample or its nearest sample are considered missing in the prediction step. The red dotted line shows where 100% accuracy lies. Source data are provided as a Source Data file.

Supplementary Figure 11. Performance of per-locus length prediction accuracy relative to GRCh38.

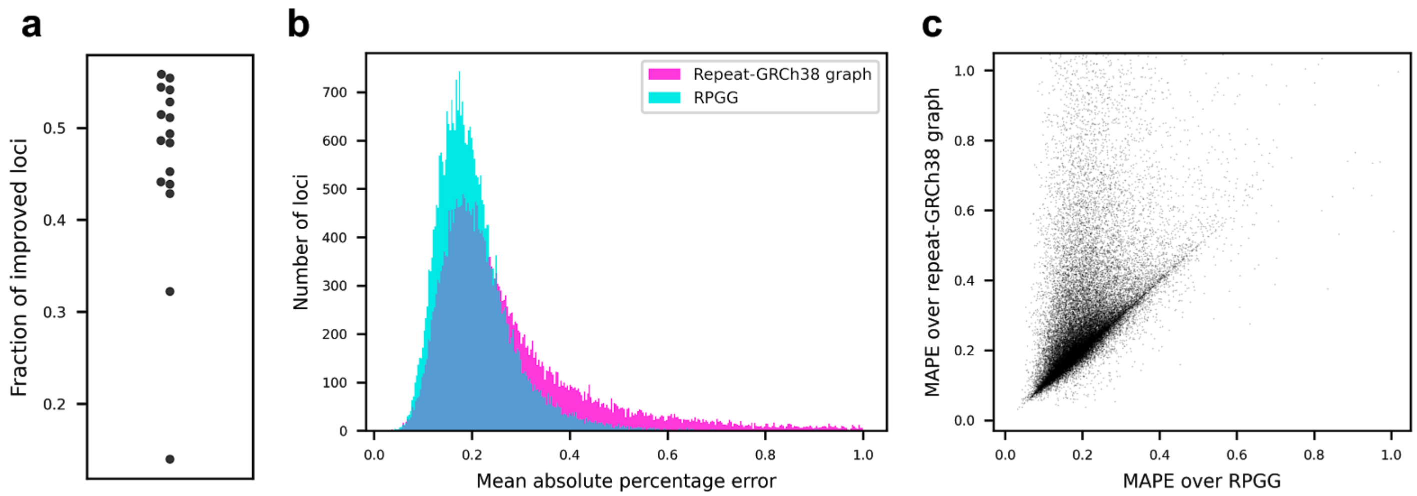

a, Fraction of loci with improved accuracy in each genome. **b,** Distribution of per-locus accuracy. Loci with MAPE greater than 1.0 are not shown. **c,** Per-locus MAPE of pangenome graphs versus hg38 graphs. Accuracy is measured by the mean absolute percentage error (MAPE) in VNTR lengths across all genomes (**b-c**). Source data are provided as a Source Data file.

Supplementary Figure 12. Correlation between the estimation error in VNTR length and in LSB.

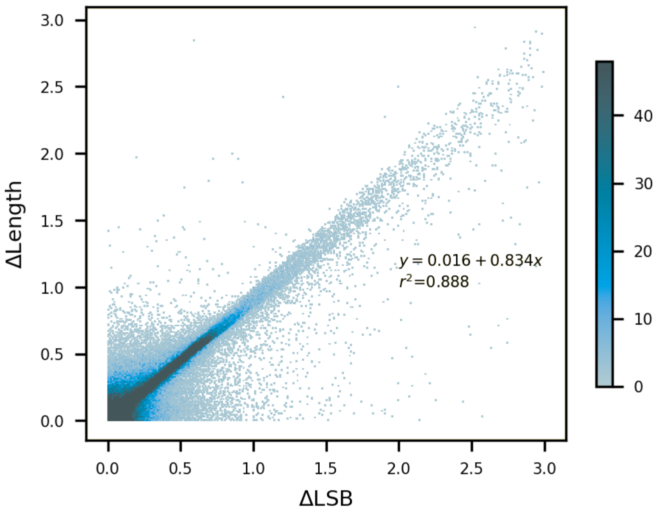

Estimation error in length was computed using absolute percentage error, i.e. $|1-gt/est|$, where *gt* is the length in assembly and *est* is the length estimated from leave-one-out analysis. Similarly, estimation error in LSB was computed as $|1-gt/est|$, where *gt* is the ground truth of the LSB for the VNTR locus (Methods) and *est* is the estimated LSB from the nearest neighbor (Methods). Data points were accumulated from 32,138 VNTR loci across 16 genomes. Source data are provided as a Source Data file.

Supplementary Figure 13. Example of deviation in LSB across samples.

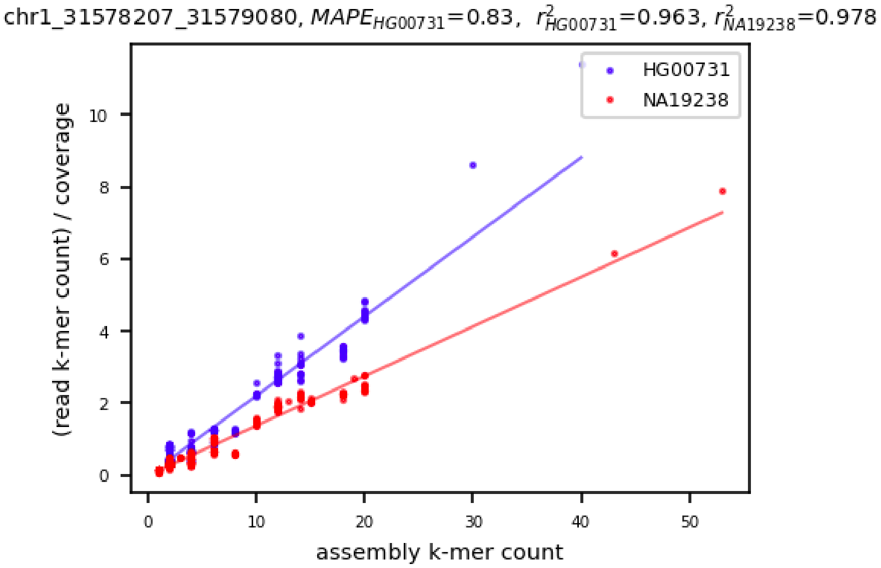

An example locus with high alignment quality but low concordance in LSB between samples. NA19238 has the most similar LSB to HG00731 based on the estimation from 397 control regions and is used to estimate the LSB of this VNTR locus in HG00731. Length prediction error is measured with mean absolute percentage error (MAPE). Source data are provided as a Source Data file.

Supplementary Figure 14. Distribution of length estimation error for loci with or without a missing haplotype.

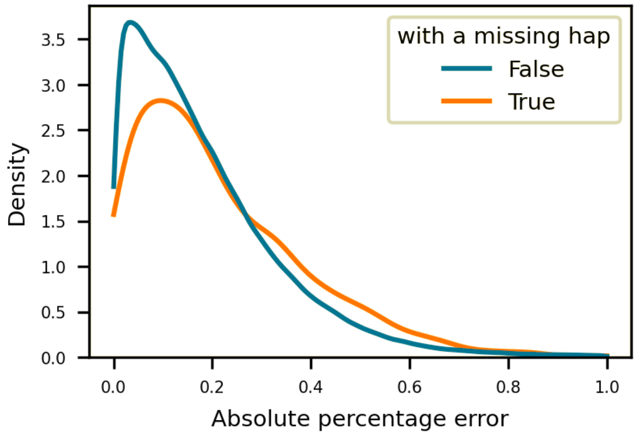

Density curves were accumulated from 32,138 VNTR loci across 16 genomes and each normalized with area 1. Source data are provided as a Source Data file.

Supplementary Figure 15. Correlation between length estimation error and fraction of novel k-mers.

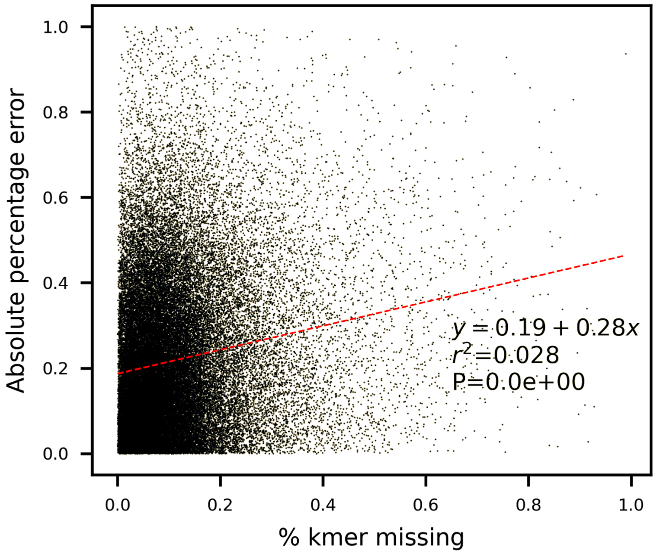

Fraction of novel *k*-mers for each locus in each genome was computed as the percentage of *k*-mers missing from the leave-one-out locus-RPGG. Data points were accumulated from 32,138 VNTR loci across 16 genomes. The P-value was derived from two-sided *t* test. Source data are provided as a Source Data file.

Supplementary Figure 16. Relationship between GC content and length prediction error.

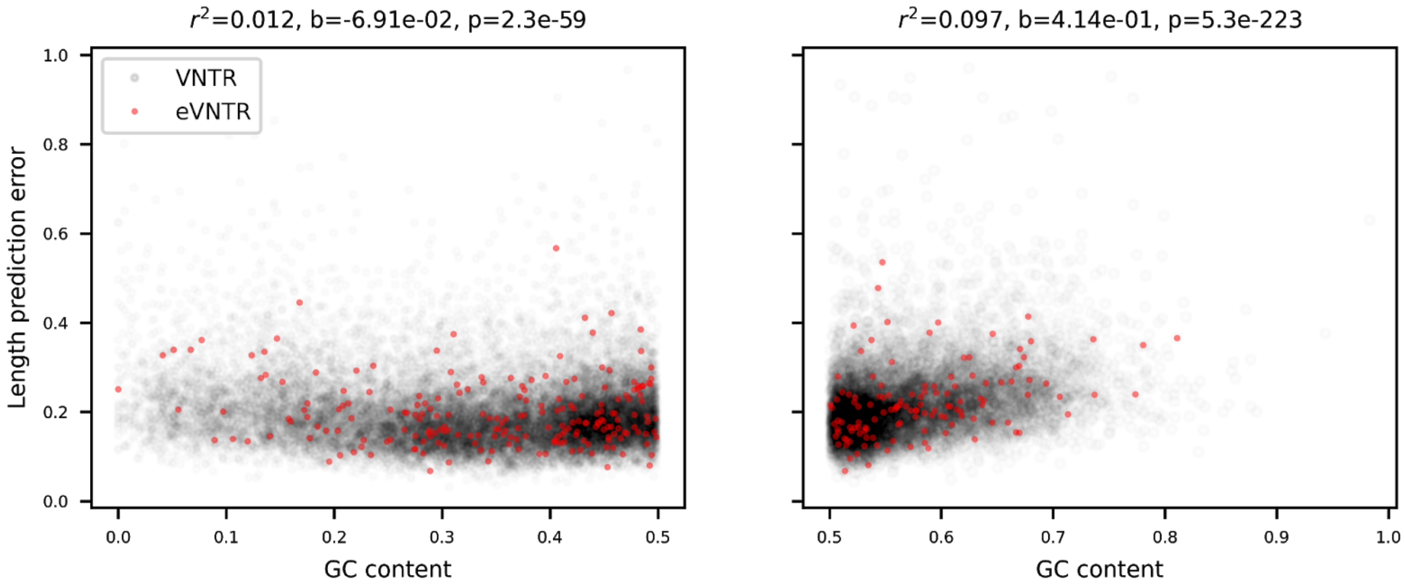

GC contents of the 32,138 VNTRs were measured on GRCh38 using bedtools nuc. Length prediction errors were measured using mean absolute percentage error in the leave-one-out analysis. The r squared, effect size and P-value (two-sided *t* test) for GC<0.5 (left) and GC>0.5 (right) are shown in the titles. Source data are provided as a Source Data file.

Supplementary Figure 17. Effect of GC content change on bias and length estimation.

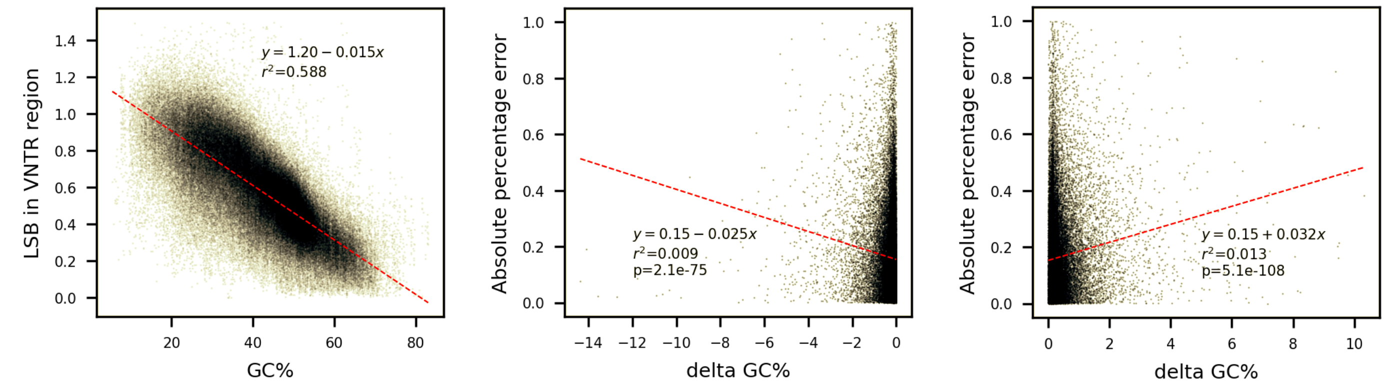

Left panel: The correlation between GC content and LSB in VNTR regions. Middle & right panels: Correlation between GC content change and length estimation error. GC content change (delta GC%) was computed from the VNTR sequence of a locus and the sequence of its nearest neighbor (same locus in another genome) in leave-one-out analysis. The analysis was restricted to HGSVC samples (HG00514, HG00733 and NA19240 trios). P-values were derived from two-sided *t* test. Source data are provided as a Source Data file.

Supplementary Figure 18. Examples of unstable loci with individuals > 10 standard deviations above the mean.

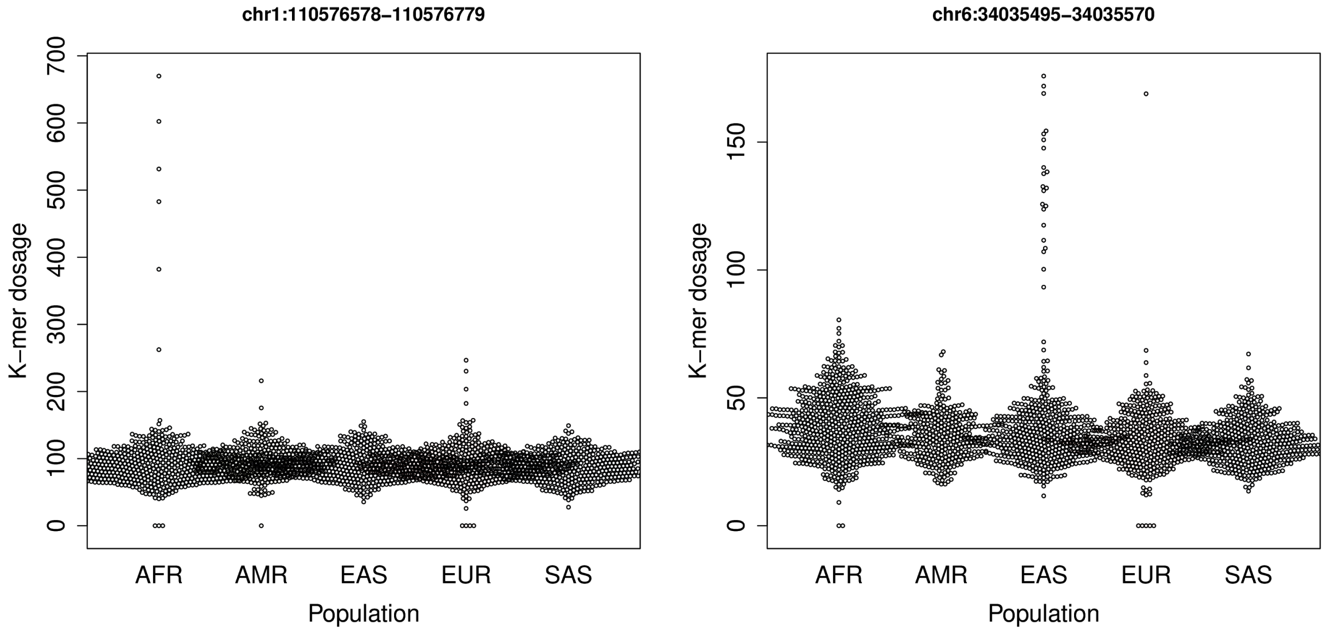

Swarm plots demonstrating highly unstable loci, determined as having an individual with coverage at least ten standard deviations above the mean. The locus on the left overlaps *KCNA2*, and the locus on the right overlaps *GRM4*. Source data are provided in Supplementary Data 3.

Supplementary Figure 19. Null and observed distributions of kmc$_d$ and r_d^2 between the EAS and AFR populations.

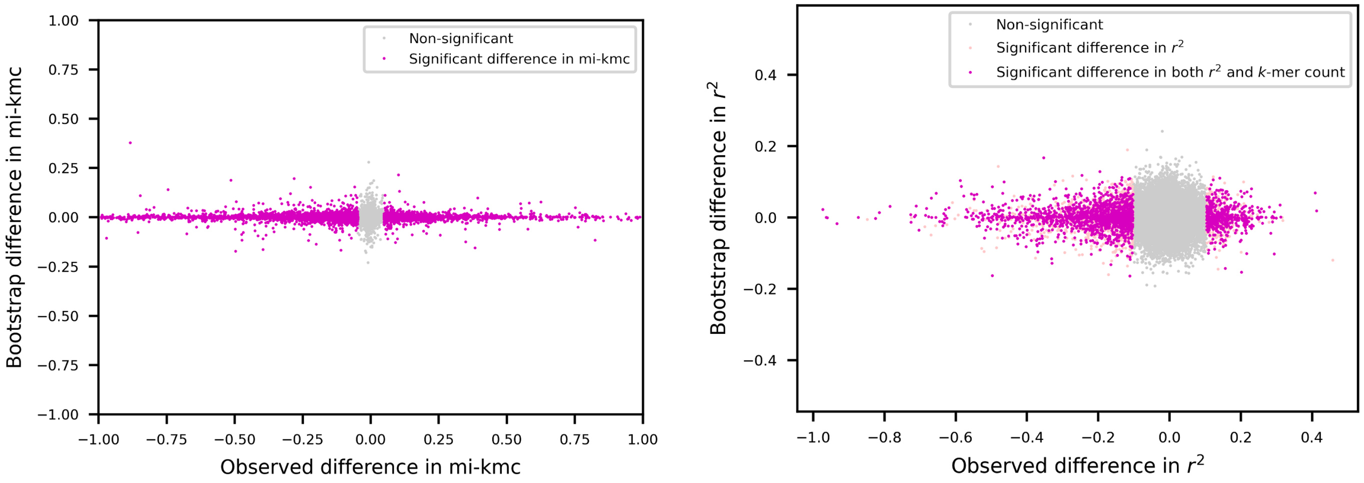

The Null distribution of difference in the count of the most informative *k*-mer (mi-kmc, left) and difference in variance explained by the most informative *k*-mer (r^2, right) at each locus was simulated using bootstrap from the EAS population with sample size matching the sum of both samples (N_{EAS}=502, N_{AFR}=661). Observed values within the two-tailed P<0.01 regions were called significant, with cutoff=(-4.702×10^{-2}, 4.834×10^{-2}) and (-1.028×10^{-1}, 1.039×10^{-1}) for mi-kmc and r^2, respectively. Source data are provided as a Source Data file.

Supplementary Figure 20. Distance of TRs and eTRs to telomere.

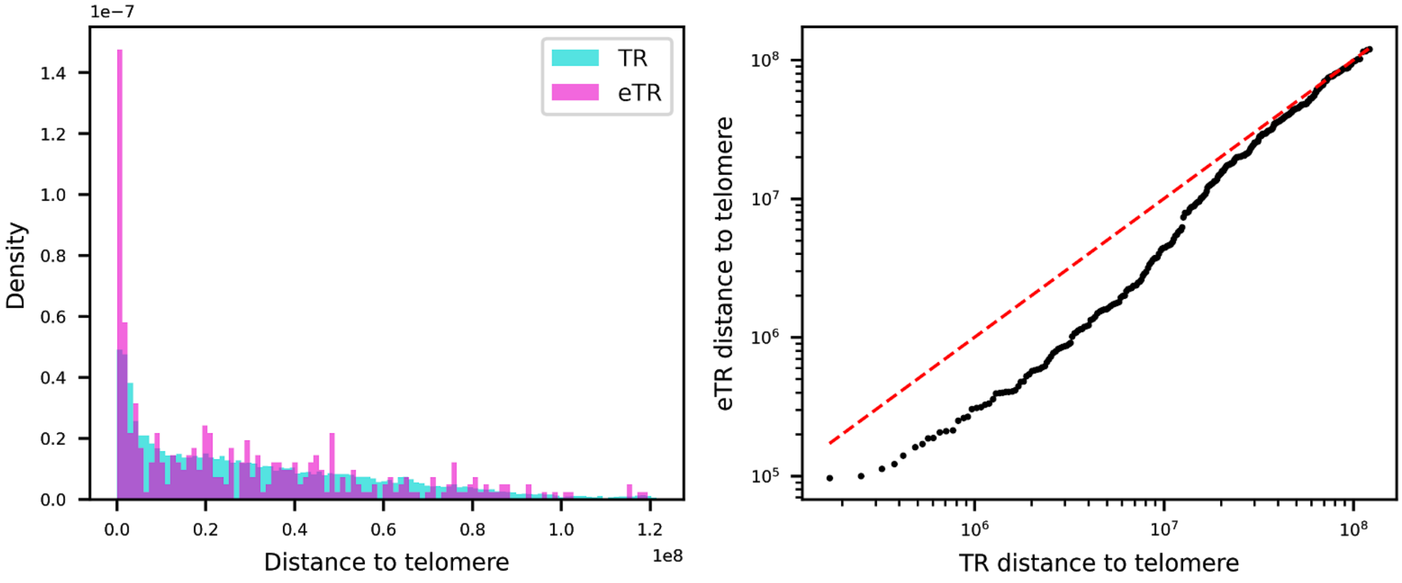

Telomere annotations were retrieved from UCSC Genome Browser and used to find the distance of a tandem repeats to its closest telomere. Distribution (left) and the q-q plot (right) of the statistics from TRs and eTRs were compared. Source data are provided as a Source Data file.

Supplementary Figure 21. Association between the top 50 pairs of eVNTR and eGene.

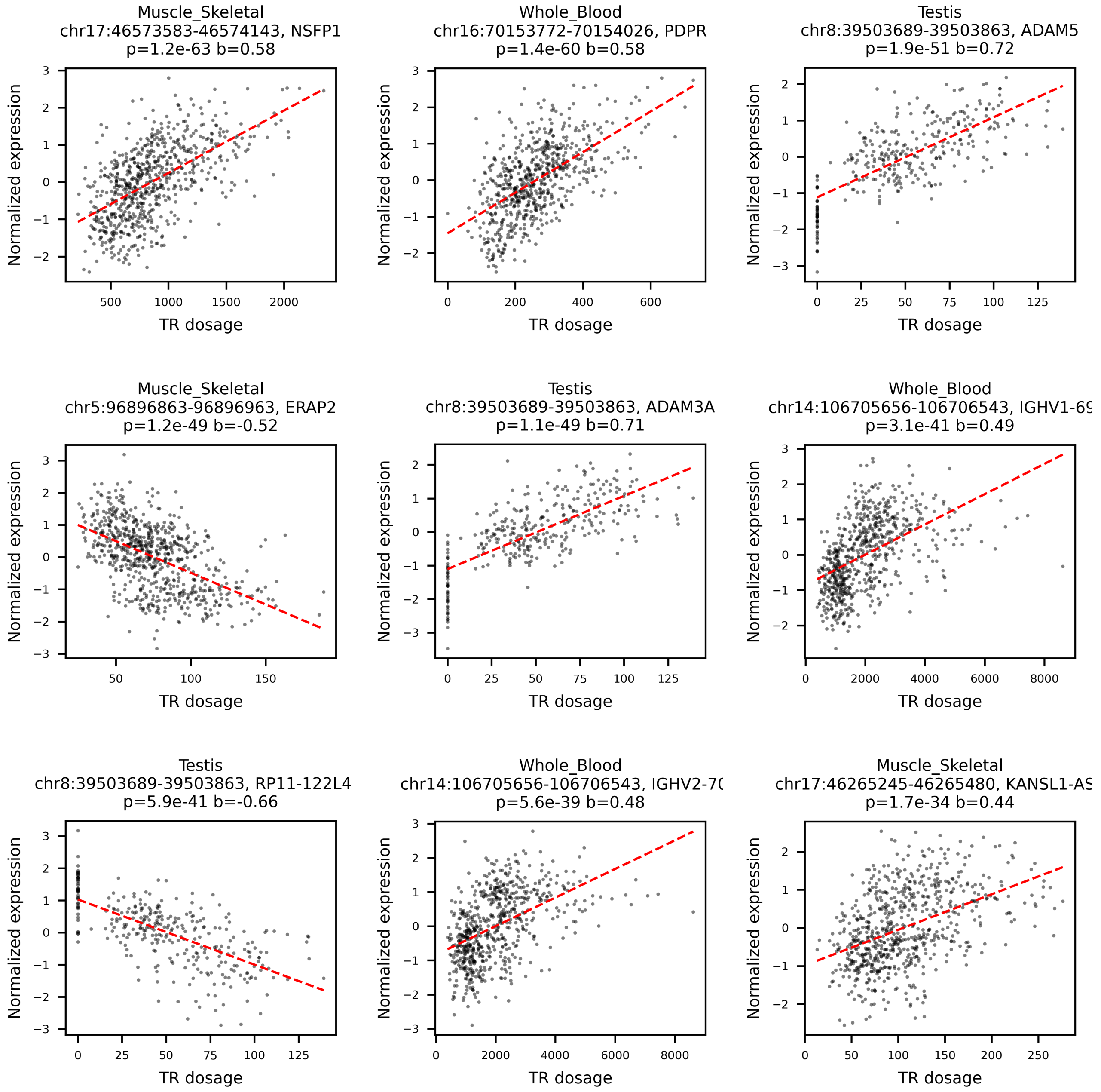

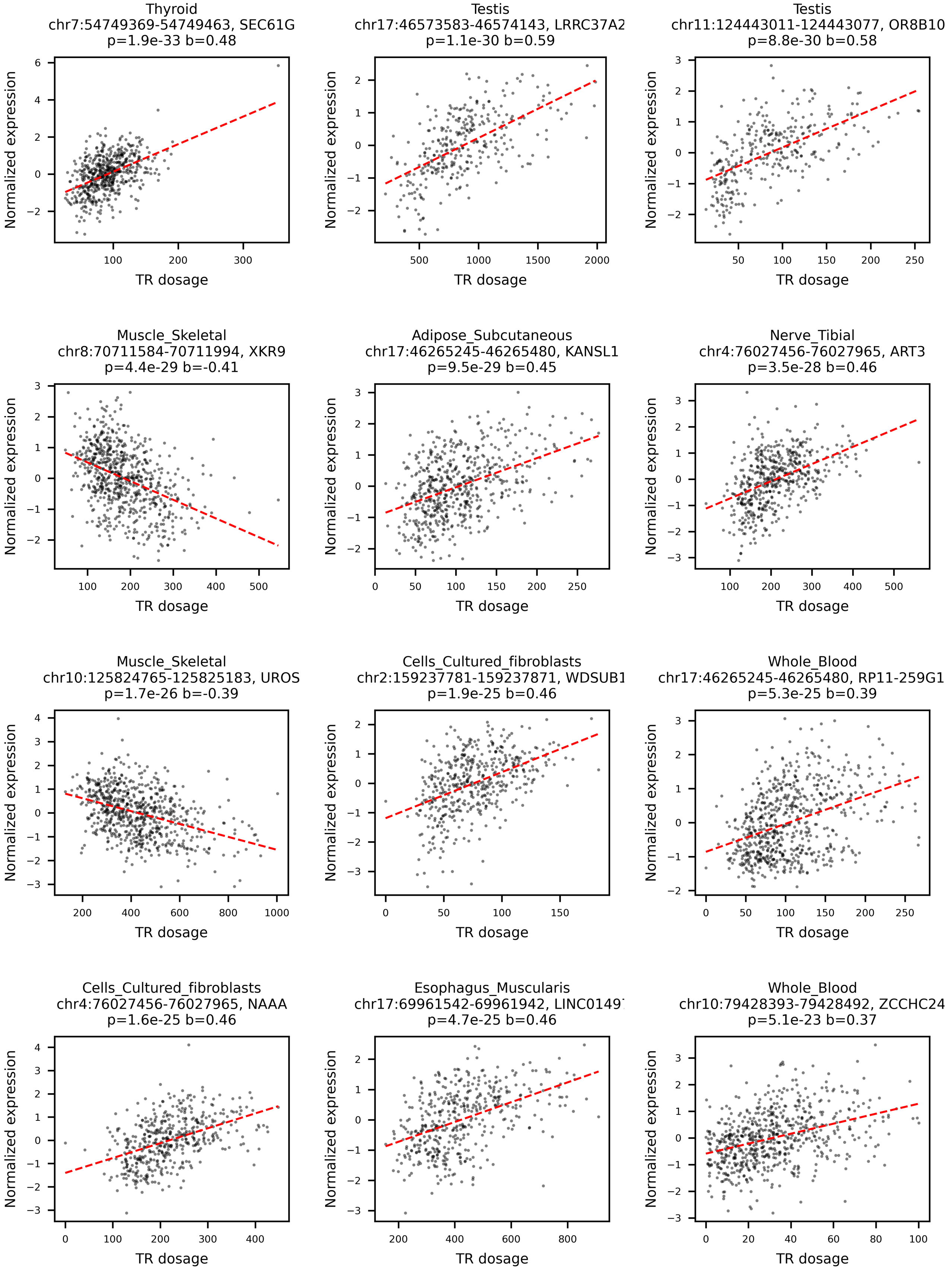
Thyroid
chr7:54749369-54749463, SEC61G
p=1.9e-33 b=0.48
Testis
chr17:46573583-46574143, LRRC37A2
p=1.1e-30 b=0.59
Testis
chr11:124443011-124443077, OR8B10
p=8.8e-30 b=0.58
Muscle_Skeletal
chr8:70711584-70711994, XKR9
p=4.4e-29 b=-0.41
Adipose_Subcutaneous
chr17:46265245-46265480, KANSL1
p=9.5e-29 b=0.45
Nerve_Tibial
chr4:76027456-76027965, ART3
p=3.5e-28 b=0.46
Muscle_Skeletal
chr10:125824765-125825183, UROS
p=1.7e-26 b=-0.39
Cells_Cultured_fibroblasts
chr2:159237781-159237871, WDSUB1
p=1.9e-25 b=0.46
Whole_Blood
chr17:46265245-46265480, RP11-259G1
p=5.3e-25 b=0.39
Cells_Cultured_fibroblasts
chr4:76027456-76027965, NAAA
p=1.6e-25 b=0.46
Esophagus_Muscularis
chr17:69961542-69961942, LINC0149
p=4.7e-25 b=0.46
Whole_Blood
chr10:79428393-79428492, ZCCHC24
p=5.1e-23 b=0.37
Normalized expression
TR dosage

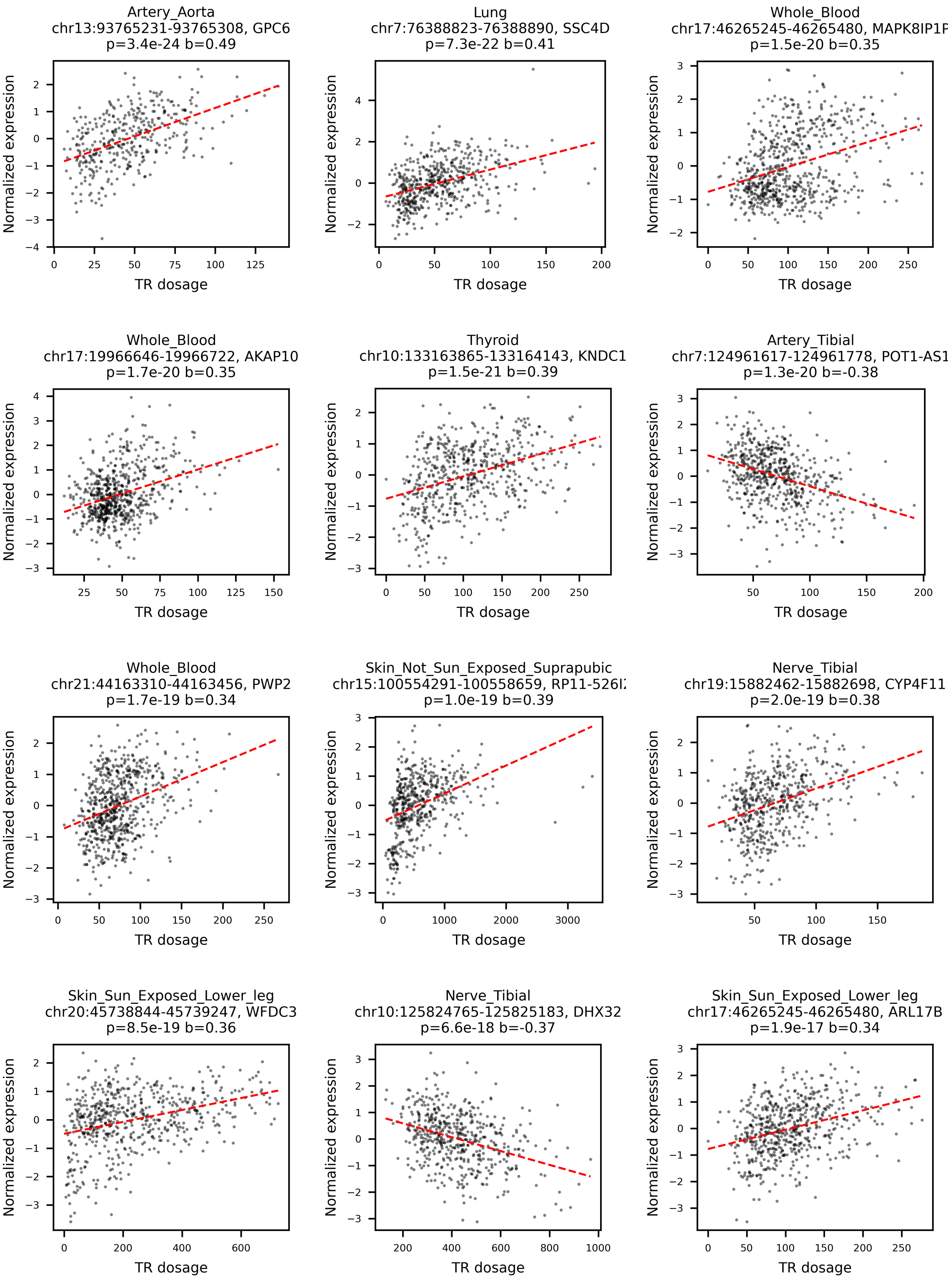
Artery_Aorta
chr13:93765231-93765308, GPC6
p=3.4e-24 b=0.49
Lung
chr7:76388823-76388890, SSC4D
p=7.3e-22 b=0.41
Whole_Blood
chr17:46265245-46265480, MAPK8IP1F
p=1.5e-20 b=0.35
Whole_Blood
chr17:19966646-19966722, AKAP10
p=1.7e-20 b=0.35
Thyroid
chr10:133163865-133164143, KNDC1
p=1.5e-21 b=0.39
Artery_Tibial
chr7:124961617-124961778, POT1-AS1
p=1.3e-20 b=-0.38
Whole_Blood
chr21:44163310-44163456, PWP2
p=1.7e-19 b=0.34
Skin_Not_Sun_Exposed_Suprapubic
chr15:100554291-100558659, RP11-526I
p=1.0e-19 b=0.39
Nerve_Tibial
chr19:15882462-15882698, CYP4F11
p=2.0e-19 b=0.38
Skin_Sun_Exposed_Lower_leg
chr20:45738844-45739247, WFDC3
p=8.5e-19 b=0.36
Nerve_Tibial
chr10:125824765-125825183, DHX32
p=6.6e-18 b=-0.37
Skin_Sun_Exposed_Lower_leg
chr17:46265245-46265480, ARL17B
p=1.9e-17 b=0.34
Normalized expression
TR dosage

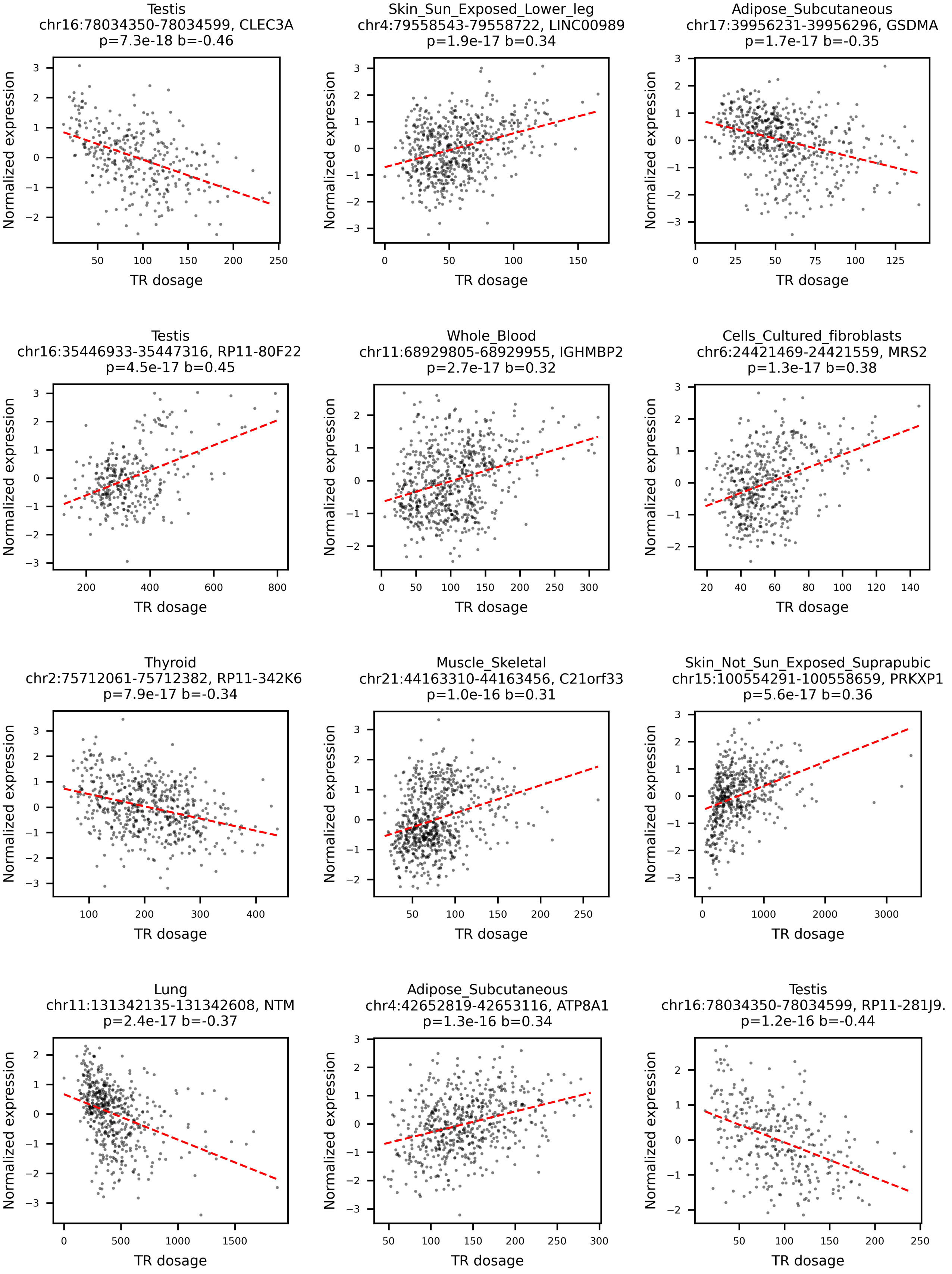
Testis
chr16:78034350-78034599, CLEC3A
p=7.3e-18 b=-0.46
Skin_Sun_Exposed_Lower_leg
chr4:79558543-79558722, LINC00989
p=1.9e-17 b=0.34
Adipose_Subcutaneous
chr17:39956231-39956296, GSDMA
p=1.7e-17 b=-0.35
Testis
chr16:35446933-35447316, RP11-80F22
p=4.5e-17 b=0.45
Whole_Blood
chr11:68929805-68929955, IGHMBP2
p=2.7e-17 b=0.32
Cells_Cultured_fibroblasts
chr6:24421469-24421559, MRS2
p=1.3e-17 b=0.38
Thyroid
chr2:75712061-75712382, RP11-342K6
p=7.9e-17 b=-0.34
Muscle_Skeletal
chr21:44163310-44163456, C21orf33
p=1.0e-16 b=0.31
Skin_Not_Sun_Exposed_Suprapubic
chr15:100554291-100558659, PRKXP1
p=5.6e-17 b=0.36
Lung
chr11:131342135-131342608, NTM
p=2.4e-17 b=-0.37
Adipose_Subcutaneous
chr4:42652819-42653116, ATP8A1
p=1.3e-16 b=0.34
Testis
chr16:78034350-78034599, RP11-281J9.
p=1.2e-16 b=-0.44
Normalized expression
TR dosage

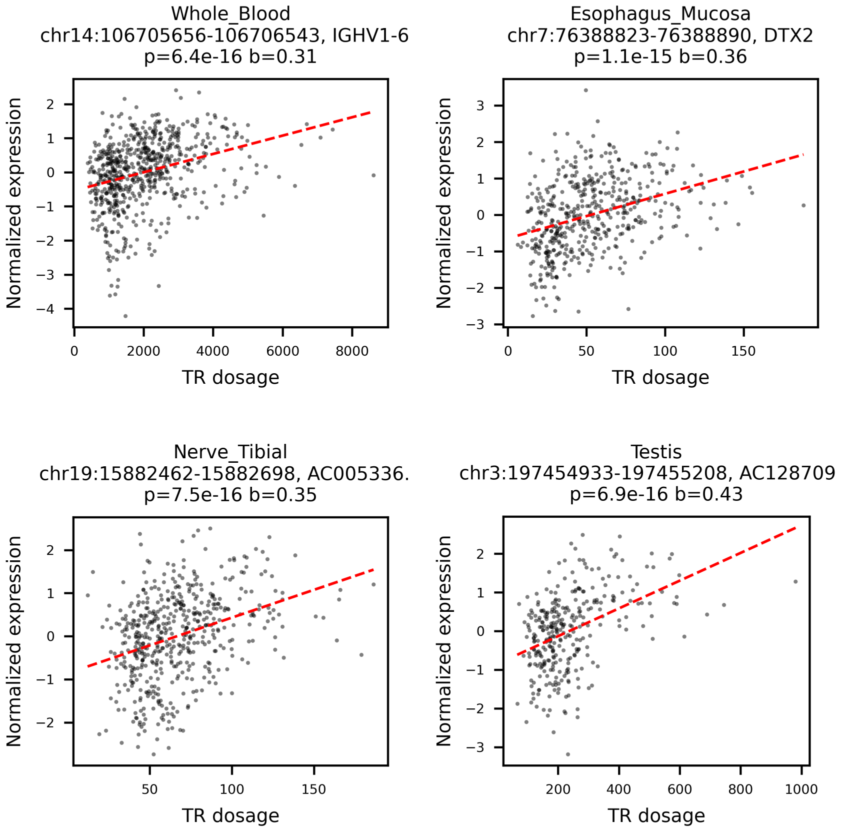

Plots are shown in order of q-value. The format of plot titles is tissue, VNTR_region, gene_name, nominal_p_val and effect_size. The linear fit is shown as a dashed red line. Nominal P-values were derived from two-sided *t* tests.

Supplementary Figure 22. Conditional association of chr5:96896863-96896963 VNTR with ERAP2 expression over chr5_96916885_T_C_b38.

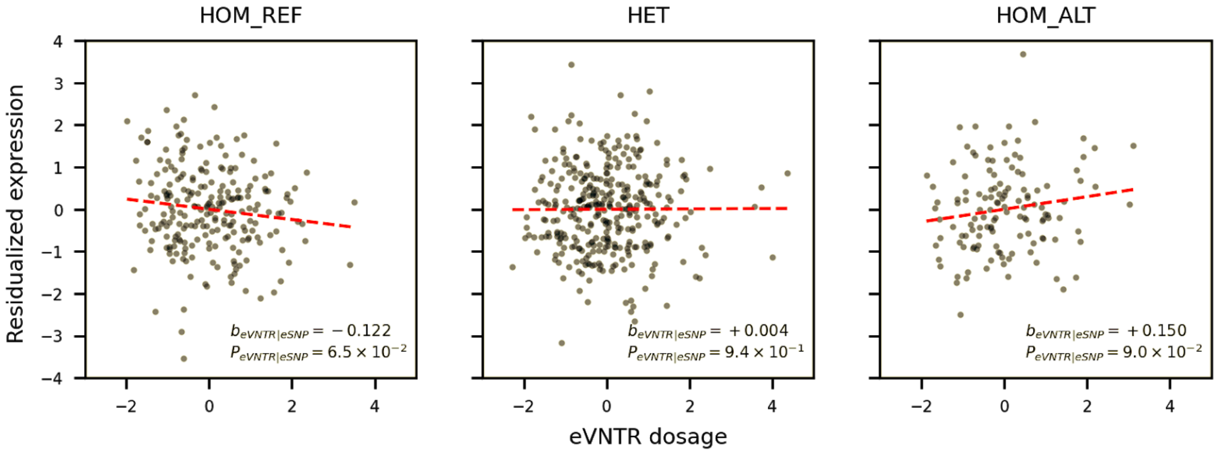

Marginal association between VNTR and expression was performed by subsetting on samples with the indicated genotype (subtitle) at the SNP site. The effect size (*b*) and P-value (*P*) for each association test (two-sided *t* test) was shown in each subpanel. The red dashed line indicates the regression line. HOM_REF, homozygous reference; HET, heterozygous; HOM_HET, homozygous alternative.

Supplementary Figure 23. Linkage disequilibrium (LD) between chr5:96896863-96896963 VNTR and nearby SNPs.

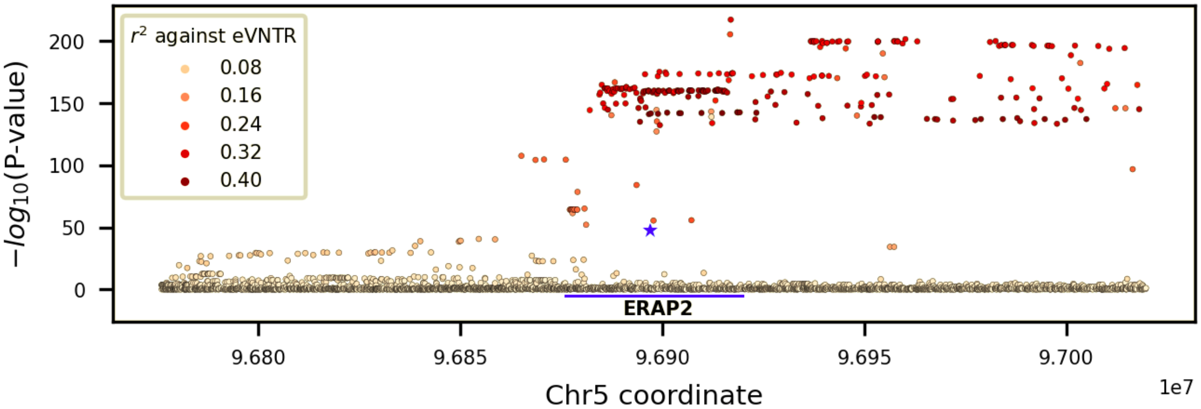

The LD between the VNTR and each nearby SNP was computed as the r^2 between genotype values. The y-axis indicates the association P-value (two-sided *t* test) with *ERAP2* expression level. The location of VNTR (blue asterisk) and ERAP2 gene (blue line) are highlighted.

Supplementary Figure 24. Spurious alignment of Illumina reads to GRCh38 at a VNTR locus.

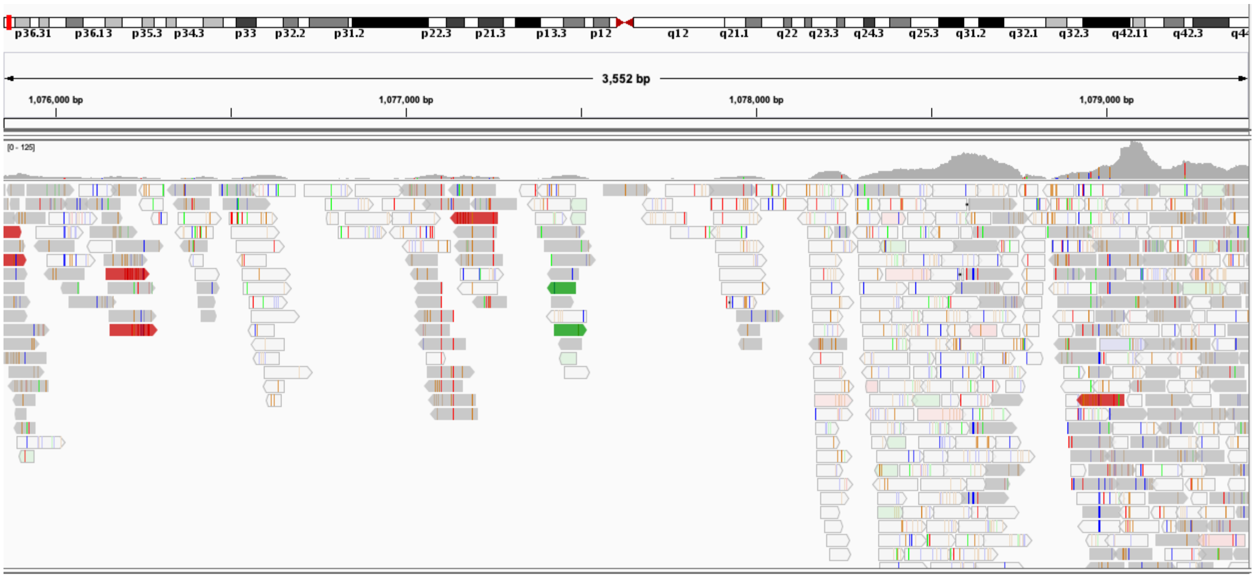

Alignment of Illumina datasets at 60x coverage from the HG00514 individual to chr1:1075852-1079425 of hg38 is visualized by Integrative Genomics Viewer (IGV)

Supplementary Figure 25. Boundary expansion recovers the proper boundary of VNTR alleles.

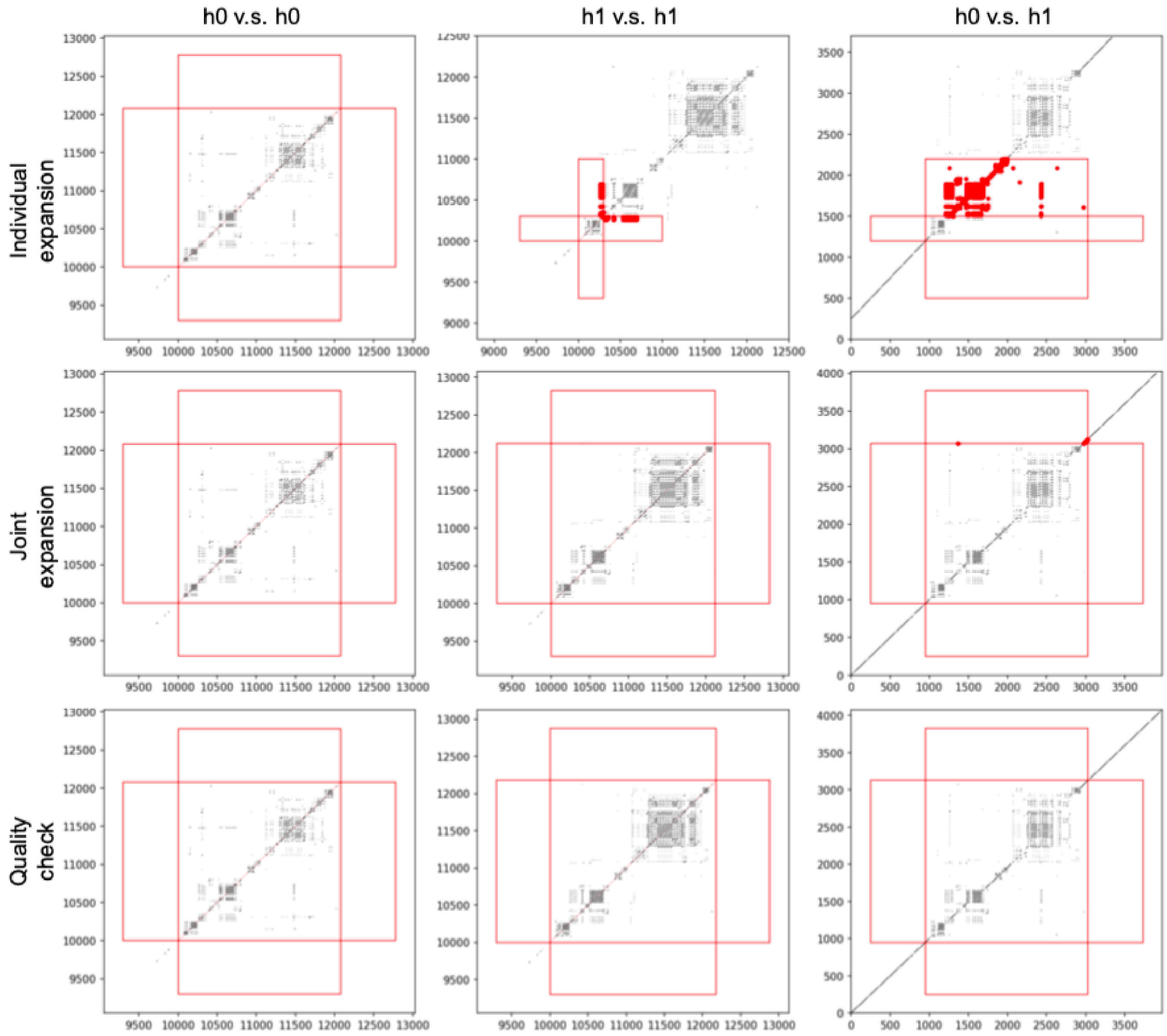

For every two VNTR alleles, the boundary expansion algorithm operates in three steps: individual expansion, joint expansion and quality check (Methods). The red boxes indicate the regions where *k*-mer matching is subject to inspection. Any matches (red dots) occurring outside of the central red box indicate the presence of shared *k*-mers between the VNTR and the flanking sequence.

Supplementary Figure 26. Distribution of number of genes overlapping shuffled high V_{ST} loci.

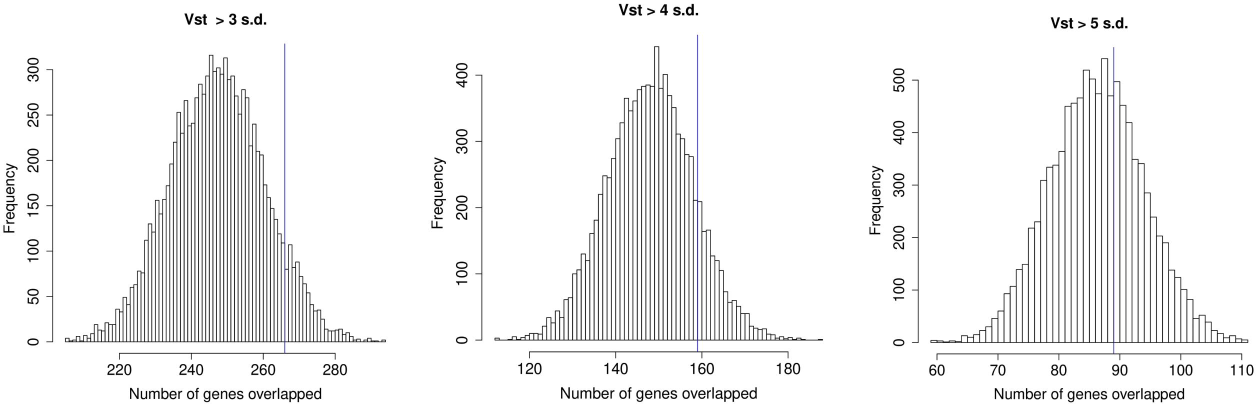

The frequency for 10,000 iterations of the number of genes overlapping high V_{ST} loci that are shuffled across the euchromatic genome. High V_{ST} are defined by a minimal number of standard deviations above the mean (3-5) (N=785, 470, and 235). The number of genes overlapping high V_{ST} loci in the original dataset are shown by the full-height vertical lines.

Supplementary Figure 27. Distribution of genes and UTR regions overlapping shuffled unstable loci.

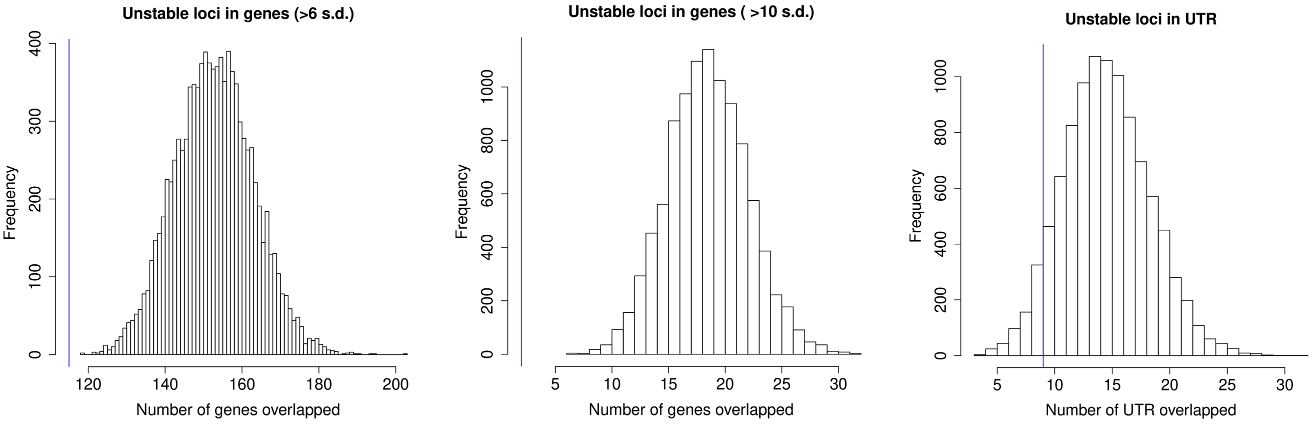

The number of genes overlapping VNTRs defined as unstable with different cutoff values: at least one individual with dosage > 6 standard deviations above the mean (N=19), and with > 10 standard deviations above the mean (N=2). The number of genes/UTRs overlapping unstable loci in the original dataset are shown by the full-height vertical lines.

Supplementary Figure 28. Number of eVNTRs shared between or specific to each tissue.

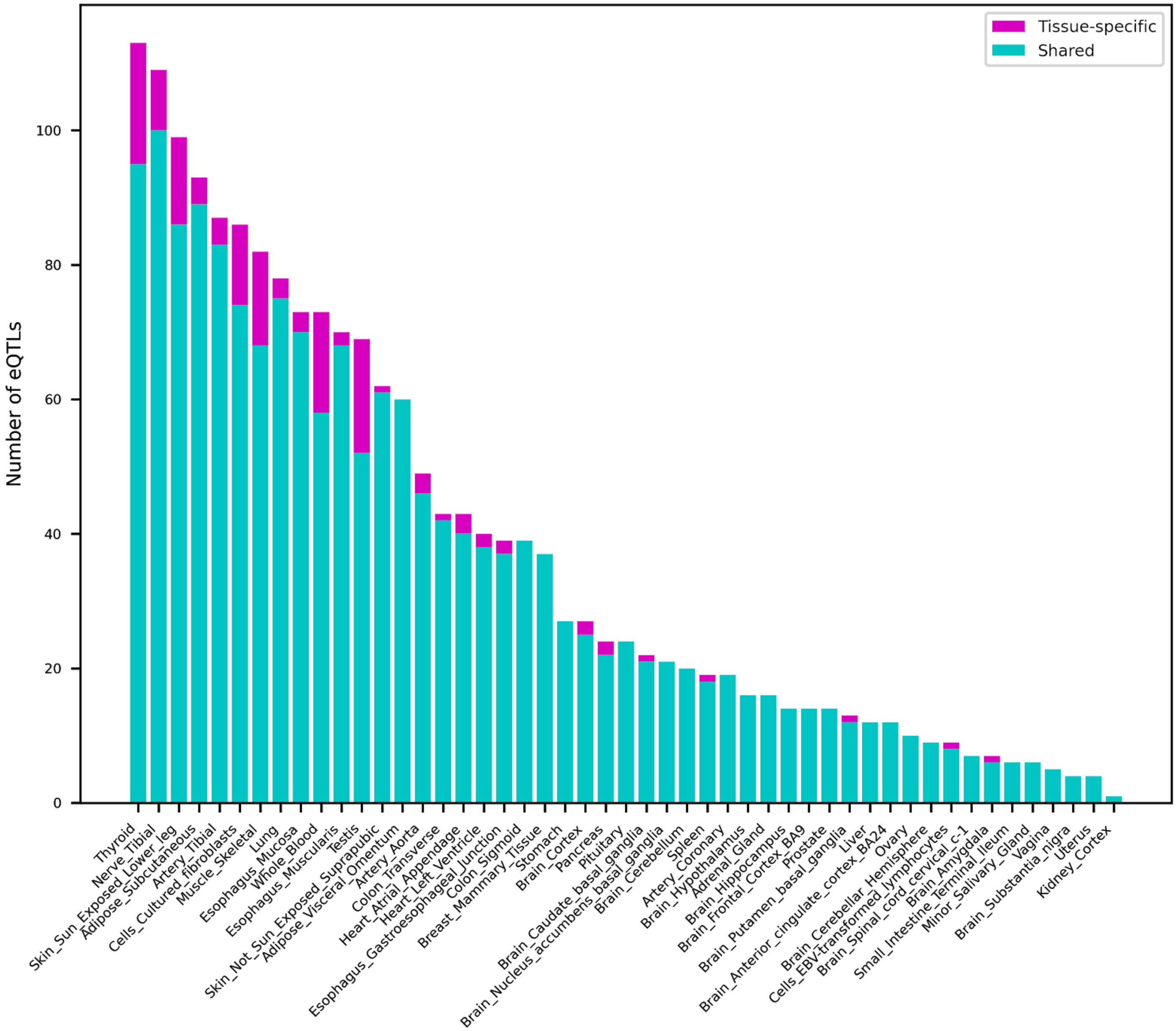

eQTL discoveries for the 32,138 VNTR loci were controlled at 5% FDR. Source data are provided in Supplementary Data 4.

Supplementary Figure 29. Length distribution of VNTRs and eVNTRs.

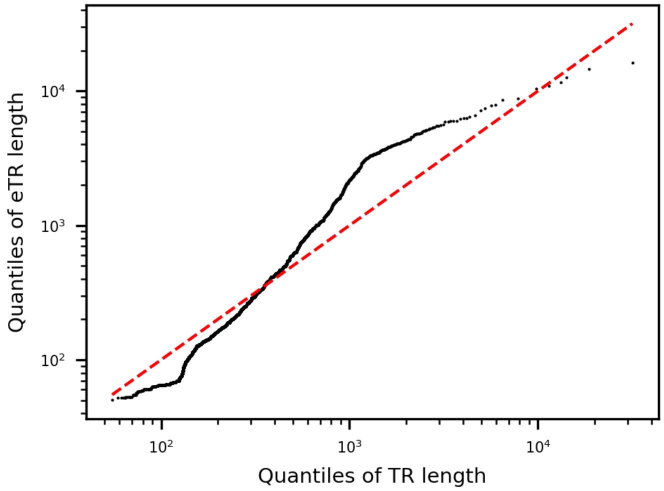

Length distribution of eVNTRs and VNTRs. eQTL discoveries for the 32,138 VNTR loci were controlled at 5% FDR. Source data are provided as a Source Data file.

Supplementary Figure 30. Sample QC on VNTR genotypes of the 1000 Genomes.

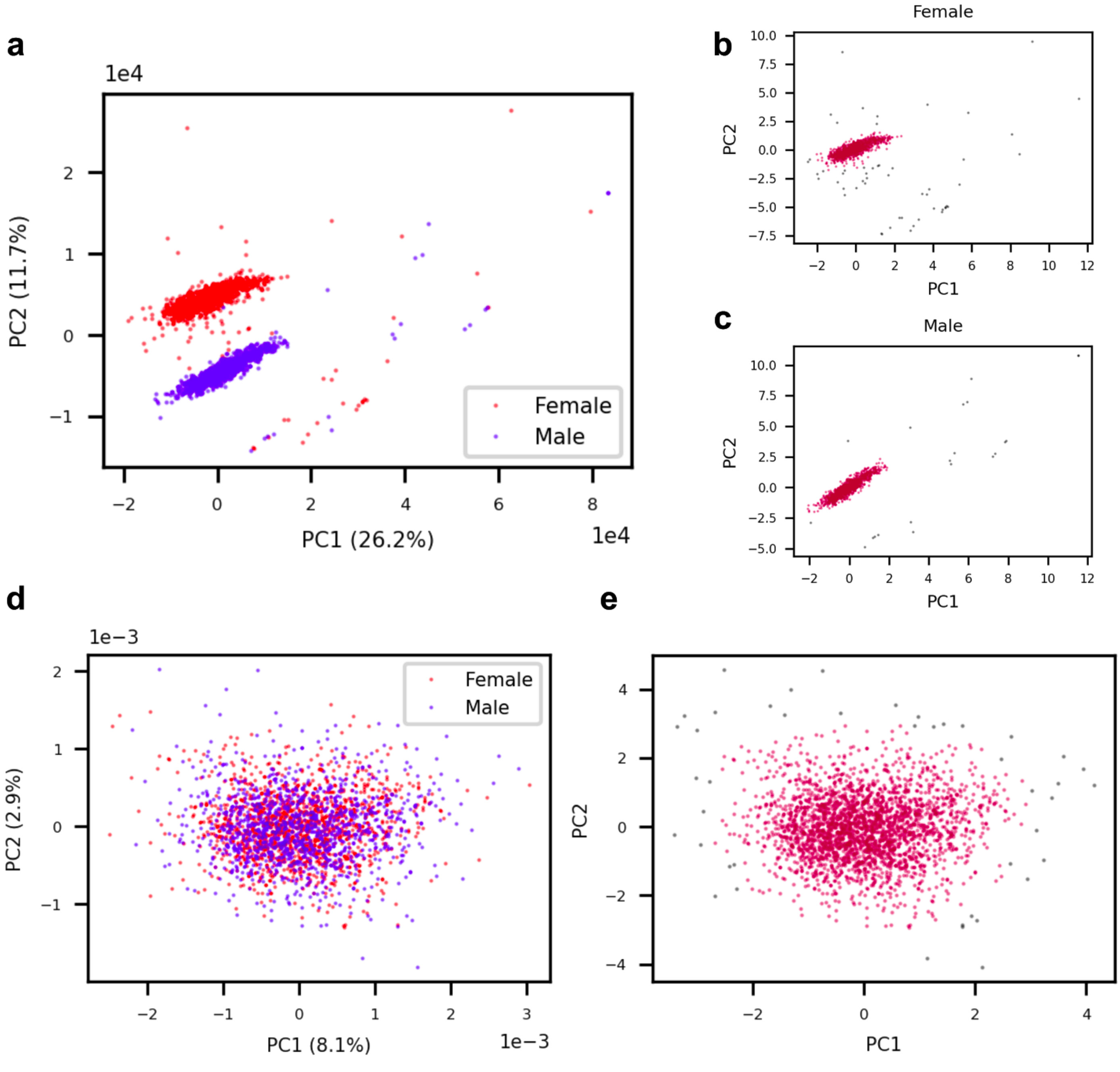

a, Joint PCA plot of samples using the *k*-mer dosage adjusted by coverage. **b-c,** Outlier detection, shown in gray, using DBSCAN with eps=0.5 on male (b) and female individuals (c). **d-e** Joint PCA plot of samples using the LSBs from 397 control regions (c) and the outliers detected using DBSCAN with eps=0.5 (d). Source data are provided in Supplementary Data 3.

Supplementary Figure 31. Sample QC on VNTR genotypes the GTEx Genomes.

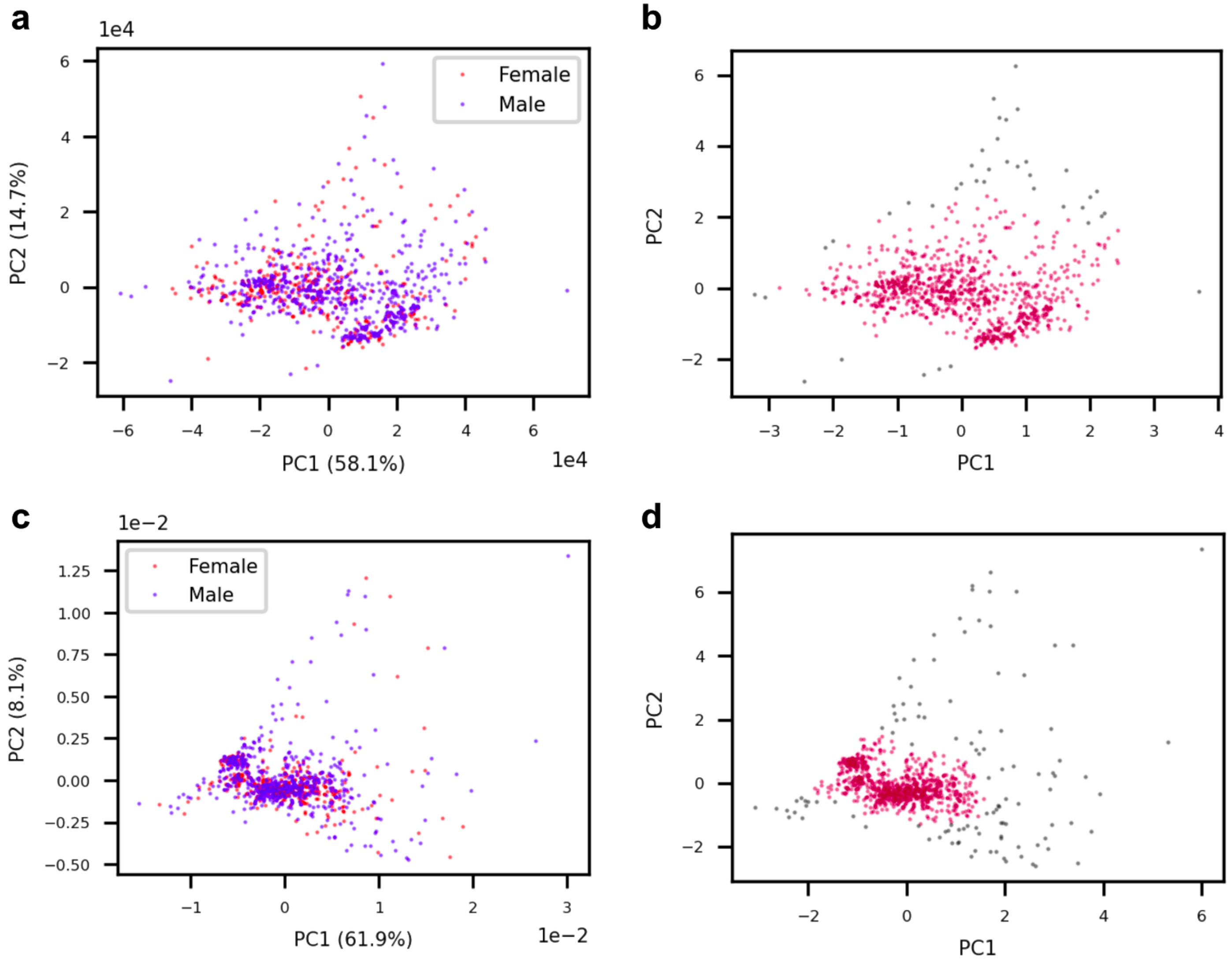

a-b Joint PCA plot of samples using the *k*-mer dosage adjusted by coverage and allelic dosage. (a) and the outliers detected, shown in gray, using DBSCAN with eps=0.5 (b). **c-d** Joint PCA plot of samples using the LSBs from 397 control regions (c) and the outliers detected using DBSCAN with eps=0.3 (d).

Supplementary Figure 32. Growth of relative VNTR-graph size.

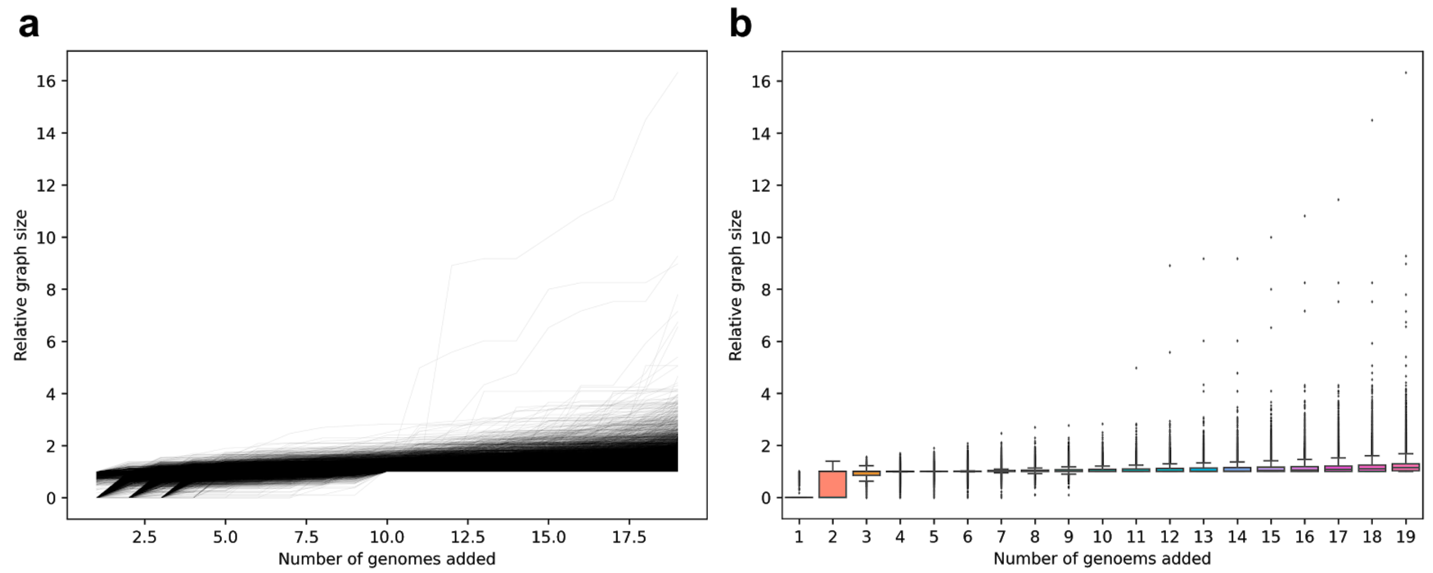

The growth curve (**a**) and the distribution of graph size (**b**) if adding genomes in an incremental manner are shown for the 32,138 VNTR loci. Relative graph size is the ratio between the number of nodes, or *k*-mers, in the RPGG and the median number of nodes in a single genome. Source data are provided as a Source Data file.

Supplementary Figure 33. Example of under-alignment of orthologous VNTR sequences by pggb.

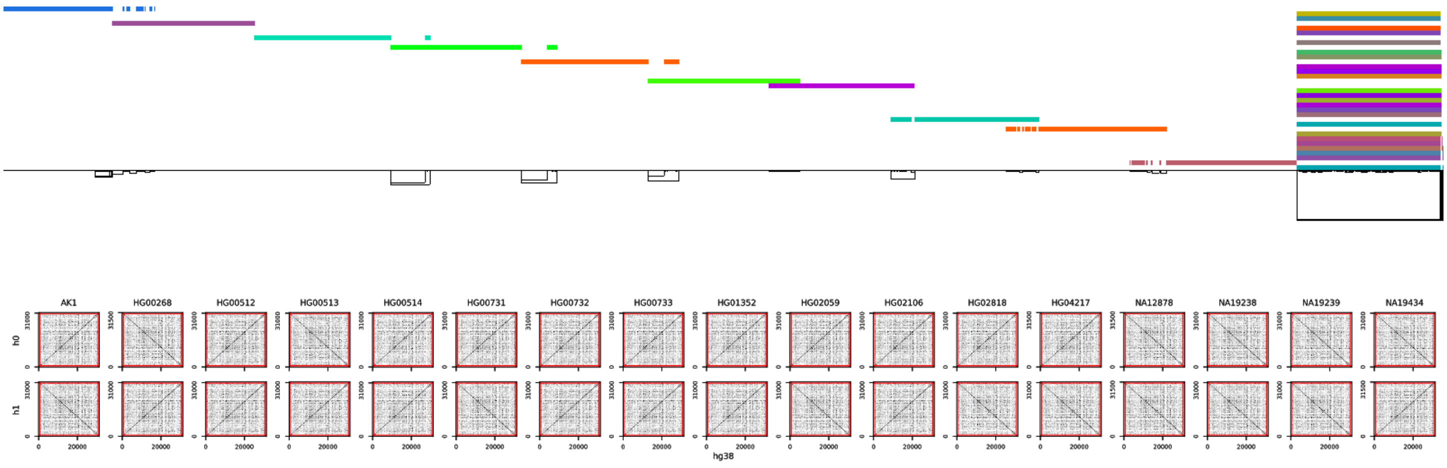

(Top) The multiple sequence alignment result of pggb for 34 VNTR haplotypes at chr12:37898555-37928455 plus 700 bp flanking sequences on each side. (Bottom) The dot plots of all haplotypes against GRCh38.

Supplementary Figure 34. Misalignment of simulated VNTR reads by bwa.

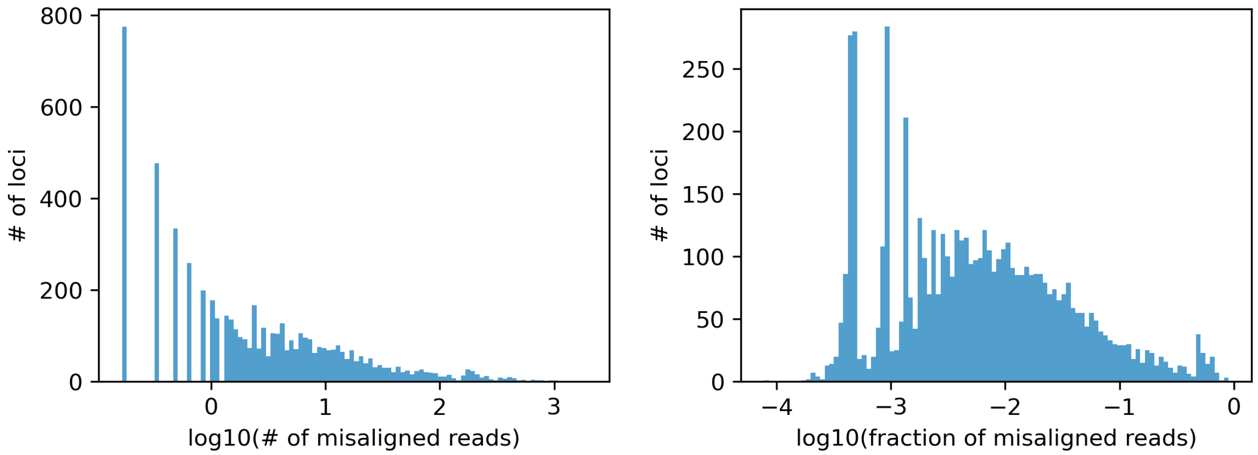

(Left) Number of misaligned VNTR reads averaged across samples. (Right) Fraction of misaligned reads averaged across samples. 32,138 VNTR loci over six genomes, including HG00512, HG00513, HG00731, HG00732, NA19238 and NA19239 were included in this experiment. Loci without misalignments are not shown for clarity. 30x error-free paired-end reads were simulated from the six genomes and each mapped to GRCh38+ALT+decoy+HLA (the hs38DH in bwa) using bwa-mem2 to follow the alignment procedures in the 1KGP and the GTEx project. We define that a read is misaligned if its location is beyond 1 kbp to the boundary of its original VNTR locus. Source data are provided as a Source Data file.

Supplementary Figure 35. Misalignment of VNTR reads to GRCh38 rescued by danbing-tk.

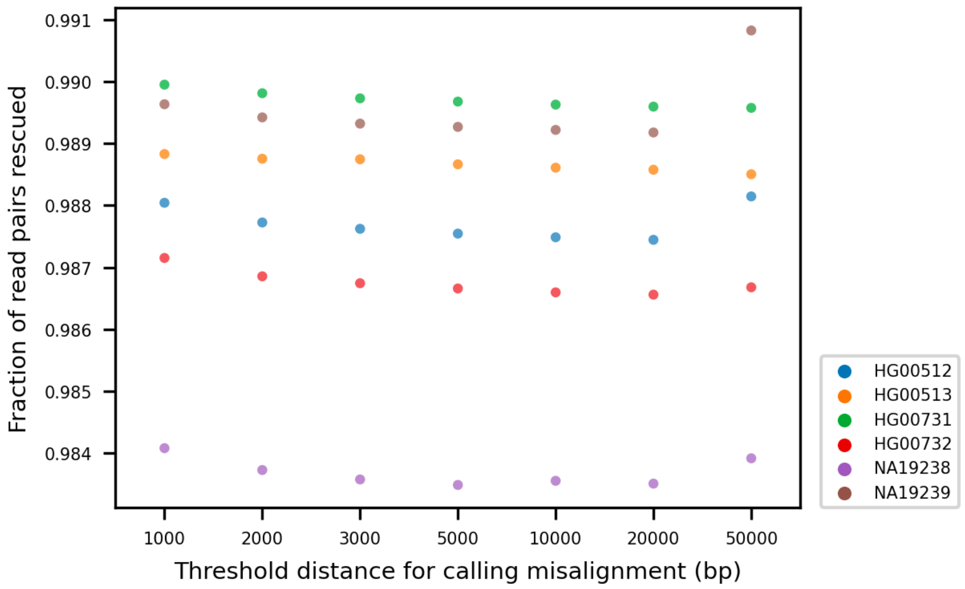

Read pairs misaligned by bwa were extracted and aligned to RPGGs using danbing-tk. A misalignment is called if the distance of any end of the read pair to its original VNTR locus is greater than the threshold. Options "-thcth 50 -cth 45 -rth 0.5" were used for danbing-tk align, same as the setting for genotyping the 1000 and the GTEx genomes. Source data are provided as a Source Data file.

Supplementary Figure 36. Relationship between VNTR length and prediction error.

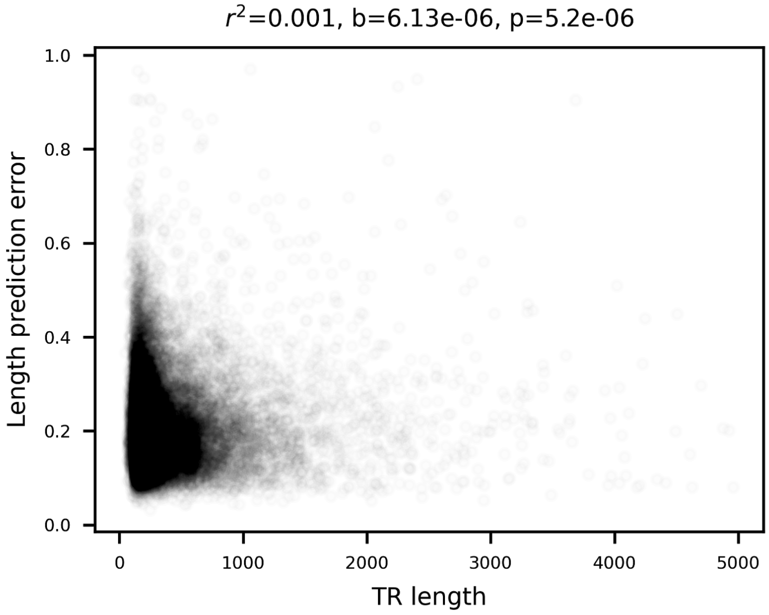

VNTR lengths of 32,138 loci were averaged across 19 genomes. Length prediction errors were measured using mean absolute percentage error in the leave-one-out analysis. The r squared, effect size and P-value (two-sided *t* test) are shown in the title. Source data are provided as a Source Data file.

Supplementary Figure 37. Relationship between eVNTR P-value and prediction error.

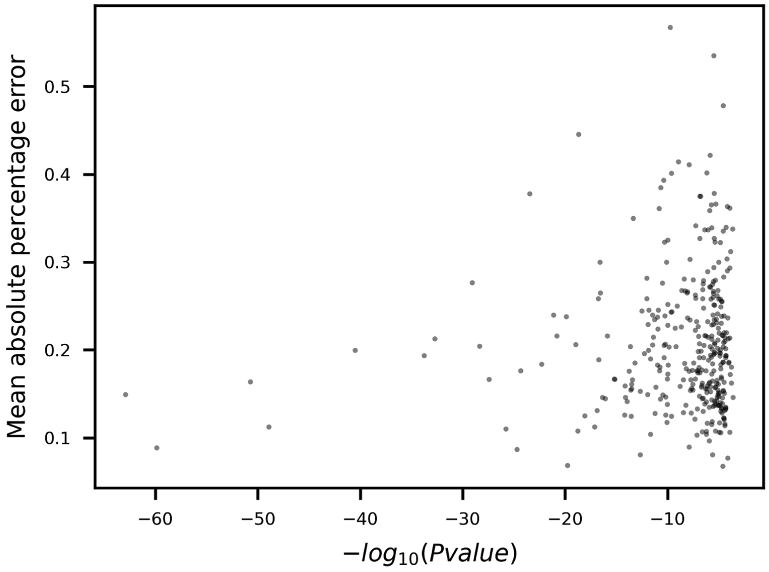

Nominal P-values (two-sided *t* test) of eVNTRs were Bonferroni-corrected. Length prediction errors were measured using mean absolute percentage error in the leave-one-out analysis. Source data are provided as a Source Data file.

Supplementary Figure 38. Comparing the alignment accuracy with and without threading.

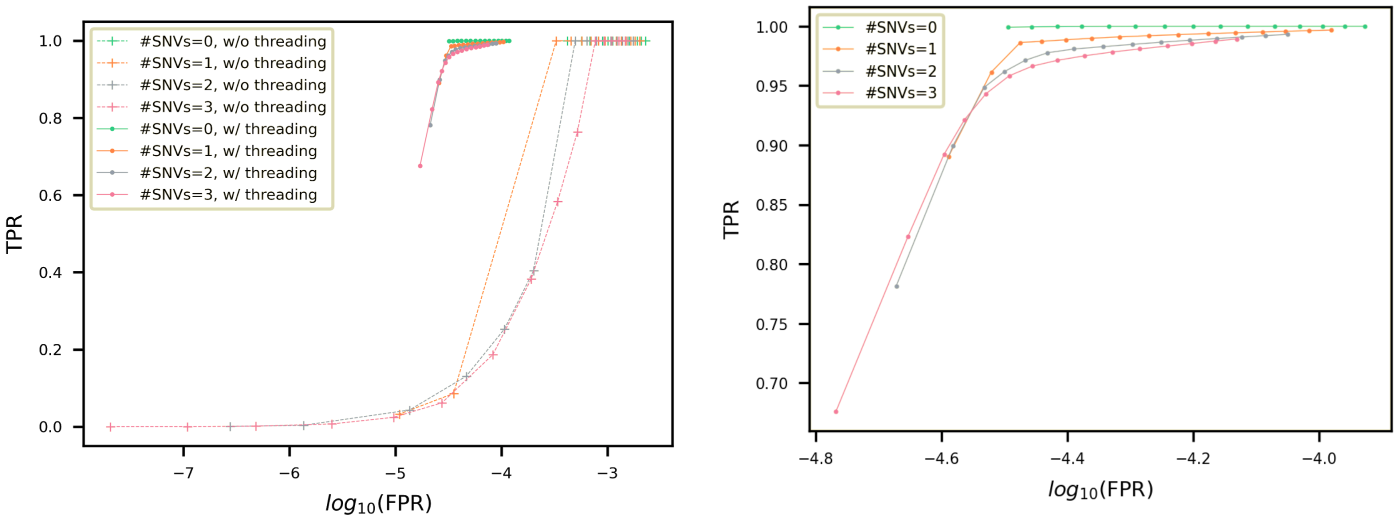

Paired-end 150 bp reads were simulated with or without SNVs and mapped to unpruned RPGG. A read is considered correctly mapped if its VNTR *k*-mers are assigned to the correct VNTR locus. Each curve is parameterized by percent identity threshold (linspace distributed between 35% and 90%). For runs with threading enabled (solid lines in both panels), cth was set to 30, and four nucleotide corrections were allowed. TPR, true positive rate; FPR, false positive rate. Source data are provided as a Source Data file.

Supplementary Figure 39. Replication of *V*st on the 698 genomes related to the 1KGP samples.

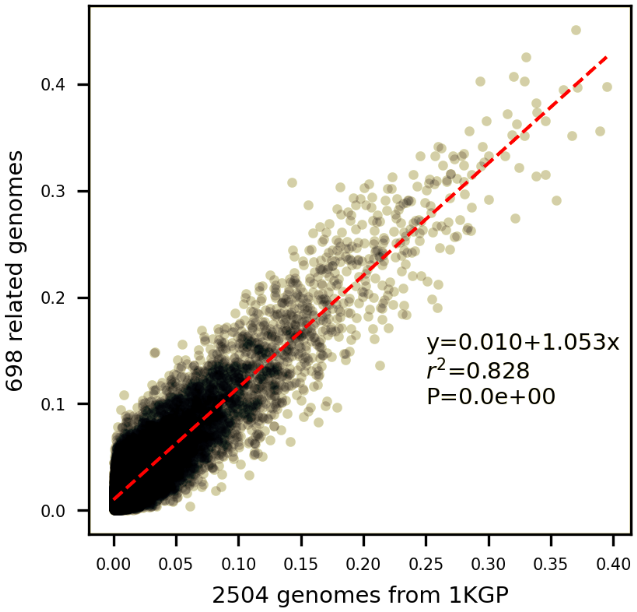

The 2,504 1KGP samples were retrieved from ENA project PRJEB31736. The 698 genomes were retrieved from ENA project PRJEB36890. *V*st was computed over the 32,138 VNTR loci using the total kmer dosage as proxy for length. The P-value was derived from two-sided *t* test. Source data are provided as a Source Data file.

Supplementary Figure 40. Incremental RPGG construction and change in boundary annotations.

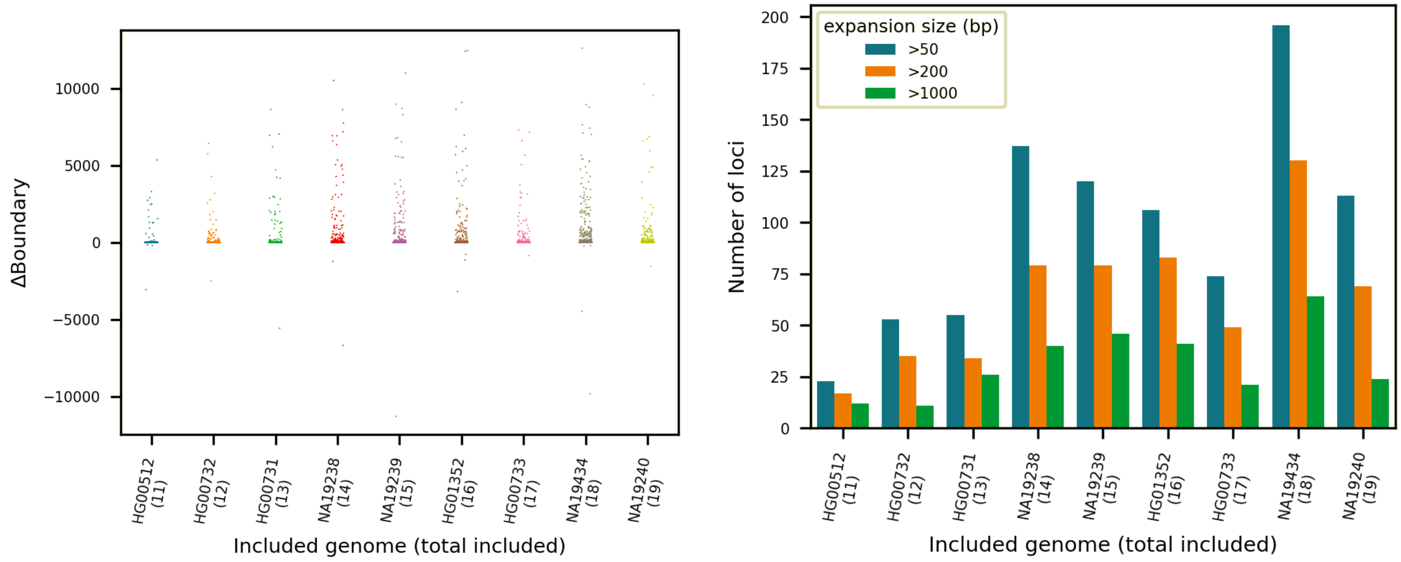

Left panel: Distribution of boundary change relative to the previous iteration of RPGG construction. Right panel: Number of loci with expansion size passing each threshold (legend) in each iteration. Δboundary is computed by summing the change in boundaries relative to the previous iteration and dividing the value by the number of supporting haplotypes. Boundary expansion was applied to the initial set of 84,411 loci annotated using TRF. Source data are provided as a Source Data file.

Supplementary Tables

Supplementary Table 1. Initial VNTR discoveries

	AK1	HG00514	HG00733	NA19240	NA24385	Pangenome
TRF	137,939	138,328	144,364	143,315	127,156	-
Boundary expansion	54,870	57,505	64,711	65,027	53,867	-
Merging						84,411

Supplementary Table 2. False mapping of reads by danbing-tk over the initial 73,582 loci.

	FP from untracked regions	Inter-locus FP	Total FP	FN***	Union of loci
HG00512	2,407	329	2,465	2,690	4,705
HG00513	2,574	336	2,643	2,614	4,827
HG00731	2,540	330	2,595	2,328	4,500
HG00732	2,781	320	2,841	3,113	5,476
NA19238	2,678	340	2,744	3,054	5,282
NA91239	2,452	342	2,520	2,832	4,855
Union of loci	5,919	497	5,999	9,525	13,800
Fraction of loci*	8.04%	0.68%	8.15%	12.94%	18.75%
Fraction removed**	71.50%	95.20%	71.60%	84.70%	78.10%

* Union of loci divided by 73,582.

** Fraction of loci in the union set with genotyping quality $r2<0.96$.

*** Unaligned reads due to graph pruning of nodes not supported by short reads.

Supplementary Table 3. eVNTRs discovered in this work that overlap with other studies

Case		1	2
This work	eVNTR.chrom	chr16	chr16
	eVNTR.start	89429084	69325358
	eVNTR.end	89430599	69325494
	eVNTR.length	1515	136
	eVNTR.eGene(s)	RP11-104N10.2	PDF,SNTB2,TERF2,NIP7
	eVNTR.beta(s)	-0.24	2.85E-14,1.66E-11,5.51E-10,2.74E-08
Fotsing et al. 2019	eSTR.chrom	chr16,chr16	
	eSTR.start	89429890,89430476	
	eSTR.end	89429901,89430493	
	eSTR.length	11,17	
	eSTR.eGene(s)	ANKRD11,ANKRD11	
	eSTR.beta(s)	-0.194,0.270	
	number of overlapping eGenes(s)	0	
Bakhtiari et al. 2018	eVNTR.chrom		chr16
	eVNTR.start		69325359
	eVNTR.end		69325495
	eVNTR.length		136
	eVNTR.eGene(s)		VPS4A
	eVNTR.pval(s)		5.43E-05
	number of overlapping eGenes(s)		0

Supplementary Table 4. Data source

Genome	Long read sequencing	Short read sequencing	Assembly
AK1	N/A	SRR3602738, SRR3602759	GCA_002009925.1
HG00268	SRX4382104	ERR251041,ERR251042	danbing-tk
HG00512	IGSR LRS	PRJEB9396	IGSR asm
HG00513	IGSR LRS	PRJEB9396	IGSR asm
HG00514	PRJNA300843	PRJEB9396	danbing-tk
HG00731	IGSR LRS	PRJEB9396	IGSR asm
HG00732	IGSR LRS	PRJEB9396	IGSR asm
HG00733	IGSR LRS	PRJEB9396	danbing-tk
HG01352	SRX2095531	SRR5571302, SRR5571303, SRR5571304, SRR5571305	danbing-tk
HG02059	SRX2537696	SRR5571333, SRR5571336, SRR5571337, SRR5571338	danbing-tk
HG02106	SRX4385796	Nationwide[1]	danbing-tk
HG02818	SRX3203304	SRR5571310, SRR5571311, SRR5571338	danbing-tk
HG04217	SRX4406292	ERR3239756, Nationwide[1]	danbing-tk
NA12878	SRX1837653	SRR3397076	danbing-tk
NA19238	IGSR LRS	PRJEB9396	IGSR asm
NA19239	IGSR LRS	PRJEB9396	IGSR asm
NA19240	IGSR LRS	PRJEB9396	danbing-tk
NA19434	SRX4118367	SRR5571360, SRR5571361	danbing-tk
NA24385	PacBio	ftp://ftp-trace.ncbi.nlm.nih.gov/giab/ftp/data/AshkenazimTrio/HG002_NA24385_son/NIST_Illumina_2x250bps/reads/	danbing-tk

[1] Nationwide sequences are available through James.Fitch@NationwideChildrens.org

Supplementary Table 5. Augmenting database with disease-related tandem repeats

Chr	Start	End	Associated gene	Associated disease	Motif	Type	Alternative name
chr12	2255791	2256090	CACNA1C	bipolar schizophrenia	(GACCCTGACCTGACT AGTTTACAATCACAC)n	intron	
chr12	63149772	63149849	AVPR1A	externalizing behavior	(GA)n(GT)n(A)n	intron	AVR
chr12	63153304	63153366	AVPR1A	externalizing behavior	(GATA)n	5UTR	RS1
chr12	63156354	63156429	AVPR1A	externalizing behavior	(CT)nTT(CT)n(GT)n	5UTR	RS3
chr3	129172568	129172736	CNBP	myotonic dystrophy 2	(CCTG)n	intron	
chr9	27573485	27573546	C9ORF72	amyotrophic lateral sclerosis	(GGGGCC)n	intron	

Supplementary Table 6. Comparison of alignment statistics between danbing-tk and GraphAligner.

	danbing-tk	GraphAligner
Read pairs mapped	258516 (99.96%)	247930 (95.9%)
Read pairs correctly mapped	257638 (99.62%)	211919 (81.9%)
Read pairs mismapped	878 (0.34%)	532 (0.21%)
Read pairs with low identity in at least one end	0 (0%)	27259 (10.5%)
Read pairs split	0 (0%)	8220 (3.2%)
Singletons	0 (0%)	8629 (3.3%)
Loci with correct read pairs	28468 (98.5%)	28405 (98.3%)

Source data are provided as a Source Data file.

Supplementary Table 7. Realignment statistics of misaligned VNTR reads from bwa.

Threshold*	1000		2000		3000		5000		10000		20000		50000	
Genome	N1**	N2***	N1	N2	N1	N2	N1	N2	N1	N2	N1	N2	N1	N2
HG00512	766	64064	766	62406	766	61890	766	61505	766	61214	766	61014	718	60570
HG00513	709	63473	696	61890	690	61307	690	60869	690	60578	690	60399	690	60020
HG00731	644	64076	637	62518	637	62017	637	61701	637	61413	637	61220	634	60814
HG00732	805	62659	805	61248	805	60732	805	60347	805	60064	805	59901	793	59533
NA19238	1066	66977	1066	65524	1066	64917	1066	64568	1057	64282	1057	64105	1022	63558
NA19239	685	66077	683	64563	683	63955	683	63640	683	63353	683	63111	575	62660

*The minimum to call misalignment for a read, i.e. the distance between actual read interval and aligned read interval

**Number of read pairs not rescued by danbing-tk

***Number of read pairs misaligned by bwa

Supplementary Notes

Supplementary Note 1. The full list of HGSVC members.

First Name	Last Name	Email	Affiliations
Aaron	wenger	awenger@pacificbiosciences.com	Pacbio
Adam	Mattson	cmattsson@bccrc.ca	BC Cancer
Alexej	Abyzov	Abyzov.Alexej@mayo.edu	Mayo Clinic
Allison	Regier	aregier@wustl.edu	Washington University
Alexej	Hastie	ahastie@bionanogenomics.com	Bionano Genomics
Ali	Bashir	ali.bashir@gmail.com	Icahn School of Medicine at Mount Sinai
Amy	Carlough	Amy.Carlough@jax.org	The Jackson Laboratory for Genomic Medicine
alvaro	Martinez Barrio	ambarrio@10xgenomics.com	10X Genomics
Anna	Basile	abasile@nygenome.org	New York Genome
Andre	Corvelo	acorvelo@nygenome.org	new York Genome
Arvis	Sulovari	arvis@uw.edu	University of Washington
Ashley	Sanders	ashley.sanders@embl.de	EMBL
Bernardo	Rodriguez martin	bmartin@embl.de	EMBL
Bob	Handsaker	handsake@broadinstitute.org	Broad Institute, Harvard Medical School
Brad	Nelson	bnelsj@uw.edu	University of Washington
Can	Alkan	calkan@gmail.com	Bilkent University
Charles	Lee	charles.lee@jax.org	The Jackson Laboratory for Genomic Medicine
Chong	Li	chong.li0001@temple.edu	Temple
Christopher	Yoon	cjyoon@wustl.edu	Washington University in St. Louis
Chunlin	Xiao	xiao2@ncbi.nlm.nih.gov	
Conner	Nodzak	cnodzak@uncc.edu	University of North Carolina at Charlotte
Daniel	Fordham	Daniel.Fordham@nanoporetech.com	Oxford Nanopore
Danny	Antaki	dantakli@ucsd.edu	UCSD
David	Porubsky	porubsky@uw.wdu	

Eoghan	Harrington	eoghan.harrington@nanoporetech.com	Oxford Nanopore
Evan	Eichler	eee@gs.washington.edu	University of Washington
Ernest	Lam	Elam@bionanogenomics.com	Bionano Genomics
Ernesto	Lowy Gallego	ernesto@ebi.ac.uk	EBI
Fabio	Navarro	Fabio.navarro@yale.edu	Yale University
Fereydoun	Hormozdiari	fhormozd@ucdavis.edu	UC Davis
Feyza	Yilmaz	feyza.yilmaz@jax.org	The Jackson Laboratory for Genomic Medicine
Gamze	Gursoy	gamze.gursoy@yale.edu	Yale
Giuseppe	Narzisi	gnarzisi@nygenome.org	New York Genome
Goo	Jun	Goo.Jun@uth.tmc.edu	Univ. of Texas Health Science Cetner Houston
Haley	Abel	abelhj@wustl.edu	Washington University in St. Louis
Han	Cao	han@bionanogenomics.com	Bionano Genomics
Harrison	Brand	HBRAND1@mgh.harvard.edu	Harvard
Ian	Fiddes	ian.fiddes@10xgenomics.com	10x Genomics
Ira	Hall	ira.hall@yale.edu	Yale
Jan	Korbel	korbel@embl.de	EMBL
Jana	Ebler	ebler@hhu.de	
Jason	Chin	jchin@pacificbiosciences.com	Pacific Bioscience
Joel	Rozowsky	ars@gersteinlab.org	Yale
Jonas	Korlach	jkorlach@pacificbiosciences.com	Pacific Bioscience
Jonathan	Sebat	jsebat@ucsd.edu	University of California San Diego
Joyce	Lee	jlee@bionanogenomics.com	Bionano Genomics
Junjie	Chen	junjie.chen2019@temple.edu	Temple
Kai	Ye	kaiye@xjtu.edu.cn	Xi'an Jiaotong University
Katy	Munson	kmiyamot@uw.edu	
Ken	Chen	kchen3@mdanderson.org	MD Anderson
Kun	Xiong	kun.xiong@yale.edu	Yale
Laura Carolyn	Smith	LSMITH66@mgh.harvard.edu	

Letu	Qingge	lqingge@uncc.edu	UNCC
Li	Guo	guoli_2016@outlook.com	Xi'Aan Jiaotong University
Li	Ding	lding@genome.wustl.edu	Washington University
Lisa	Brooks	brooksl@mail.nih.gov	NIH/NHGRI
Madhusudan	Gujral	mgujral@ucsd.edu	University of California San Diego
Maggi		maggic@uab.edu	UAB School of Medicine - Birmingham, AL
Marc Jan	Bonder	m.bonder@dkfz-heidelberg.de	
Mark	Gerstein	mark@gersteinlab.org	Yale
Mark	Batzer	mbatzer@lsu.edu	Louisiana State University
Mark	Chaisson	mchaisso@usc.edu	University Southern California
Marta	Byrska-Bishop	mbyrska-bishop@nygenome.org	New York Genome
Matthew	Wyczalkowski	m.wyczalkowski@wustl.edu	Washington University in St. Louis
Mike	Smith	mike.smith@embl.de	EMBL
Mike	Zody	mczody@nygenome.org	NY Genome
Michael	Schnall-Levin	mike@10xgenomics.com	10x Genomics
Mike	Talkowski	talkowski@chgr.mgh.harvard.edu	Harvard Medical School, Broad Institute, Mass. General
Miriam	Konkel	mkonkel@clemson.edu	Clemson
Nelson	Chuang	nchuang@umaryland.edu	University of Maryland
Nina	Habermann	nina.habermann@embl.de	EMBL
Omar	Shanta	oshanta@eng.ucsd.edu	UCSD
Oscar	Rodgriguez	Oscar.Rodriguez@icahn.mssm.edu	Icahn School of Medicine at Mount Sinai
Paul	Flicek	flicek@ebi.ac.uk	EMBL-EBI
Peter	Audano	paudano@uw.edu	Univeristy of Washington
Peter	Ebert	pebert@mpi-inf.mpg.de	Max Plank
Patrick	Marks	patrick@10xgenomics.com	10x Genomics
Peter	Lansdorp	plansdor@bccrc.ca	University of British Columbia
Qihui	Zhu	qihui.zhu@jax.org	The Jackson Laboratory for Genomic Medicine
Rajeeva	Musunuri	rmusunuri@nygenome.org	

Rebecca	Serra	rebecca.serra@mari@hhu.de	
Robel	Dagnow	rdagnew@usc.edu	USC
Ryan	Collins	rcollins@chgr.mgh.harvard.edu	Harvard Medical School
Ryan	Mills	remills@umich.edu	University of Michigan
Sascha	Meiers	sascha.meiers@embl.de	EMBL Heidelberg
Scott	Devine	sdevine@som.umaryland.edu	University of Maryland
Serhat	Tetikol	serhat.tetikol@sbgenomics.com	Seven Bridges
Shamoni	Maheshwari	shamoni.maheshwari@10xgenomics.com	10X Genomics
Shantao	Li	shantao.li@yale.edu	Yale
Steve	Sherry	sherry@ncbi.nlm.nih.gov	NCBI
Susan	Fairley	fairley@ebi.ac.uk	EMBL-EBI
Sushant	Kumar	sushant.kumar@yale.edu	Yale University
Tobias	Marschall	tobias.marschall@hhu.de	Heinrich Heine University Dusseldorf
Timur	Galeev	timur.galeev@yale.edu	Yale
Tobias	Rausch	rausch@embl.de	EMBL
Tonia	Brown	tjbrown@u.washington.edu	Univeristy of Washington
Uday	Shanker Evani	usevani@nygenome.org	New York Genome
Vincent	Hanlon	vhanlon@bccrc.ca	
Virginia	Nunez-Mir	nunezmir@usc.edu	USC
Wan-Ping	Lee	Wan-Ping.Lee@Pennmedicine.upenn.edu	
Wayne	Clark	wclarke@nygenome.org	New York Genome
Weichen	Zhou	arthurz@med.umich.edu	University of Michigan
Wen-Wei	Liao	wen-wei.liao@wustl.edu	Wash. University
William	Harvey	wharvey@uw.edu	Univeristy of Washington
Wolfram	Hoeps	wolfram.hoeps@embl.de	EMBL
Xian	Fan	xianfan.jhu@gmail.com	
Xinghua Mindy	Shi	mindyshi@temple.edu	Temple
Xiaofei	Yang	xfyang@xjtu.edu.cn	Xi'an Jiaotong University
Xuefang	Zhao	XZHAO12@mgh.harvard.edu	Harvard

Yang	Li	yangili1@uchicago.edu	
Zechen	Chong	zchong@uabmc.edu	UAB School of Medicine - Birmingham, AL
Zeid	Hamadeh	zhamadeh@bccrc.ca	
Zev	Kronenberg	zevk@u.washington.edu	University of Washington
