# Supplementary material for: Profiling variable-number tandem repeat variation across populations using repeat-pangenome graphs
Source: Nat Commun. 2021 Jul 12;12:4250. doi: 10.1038/s41467-021-24378-0 (PMC8275641; doi:10.1038/s41467-021-24378-0)
Supplement: Supplementary file 3 — Description of Additional Supplementary Files [file 41467_2021_24378_MOESM3_ESM.pdf]

Description of Additional Supplementary Files

File Name: Supplementary Data 1
Description: Sequencing and assembly of reference genomes

File Name: Supplementary Data 2
Description: VNTR boundary annotations for 73k loci

File Name: Supplementary Data 3
Description: Functional and disease relevance of unstable loci

File Name: Supplementary Data 4
Description: eVNTR discoveries over 32k VNTR loci

File Name: Supplementary Data 5
Description: eVNTR discoveries over 73k VNTR loci

File Name: Supplementary Data 6
Description: eVNTR discoveries README

File Name: Supplementary Data 7
Description: VNTR statistics (32k)

File Name: Supplementary Data 8
Description: VNTR statistics (73k)

File Name: Supplementary Data 9
Description: VNTR statistics README
