# Supplementary material for: Profiling variable-number tandem repeat variation across populations using repeat-pangenome graphs
Source: Nat Commun. 2021 Jul 12;12:4250. doi: 10.1038/s41467-021-24378-0 (PMC8275641; doi:10.1038/s41467-021-24378-0)
Supplement: Supplementary file 9 — Supplementary Data 6 [file 41467_2021_24378_MOESM9_ESM.pdf]

tissue:	Tissue of which the association between TR length and expression TPM count is significant
gene_id:	GENCODE/Ensembl gene ID
gene_name:	GENCODE gene name
chr:	Chromosome (gene)
start:	Gene start position
end:	Gene end position
num_var:	Number of TRs in cis-window (100kbp)
TR_chr:	Chromosome (TR)
TR_start:	TR start position
TR_end:	TR end position
TR_locus:	TR index in our database
eTR:	Is an eTR after controlling gene-level FDR from lead_TRs at 5%
lead_TR:	Is a lead_TR in the cis-window
lead_p_nominal:	Nominal P value of the lead TR in this cis-window
p_nominal:	Nominal P value of this TR
slope:	Regression slope
slope_se:	Standard error of the regression slope
qval:	Benjamini-Hochberg q-value dereived from Bonferroni-adjusted P value
