# Supplementary material for: Profiling variable-number tandem repeat variation across populations using repeat-pangenome graphs
Source: Nat Commun. 2021 Jul 12;12:4250. doi: 10.1038/s41467-021-24378-0 (PMC8275641; doi:10.1038/s41467-021-24378-0)
Supplement: Supplementary file 12 — Supplementary Data 9 [file 41467_2021_24378_MOESM12_ESM.pdf]

chr:	Chromosome where the VNTR is annotated
start:	Start coordinate of the VNTR in hg38
end:	End coordinate of the VNTR in hg38
ref.len:	VNTR length in hg38 after boundary expansion
asm.len:	Mean VNTR length across assemblies after boundary expansion
chr:	Chromosome where the VNTR is annotated
asm.len.std:	Standard deviation of VNTR lengths across assemblies after boundary expansion
asm.n:	Number of supporting genomes beyond hg38
hg38.acc:	Genotyping accuracy using repeat-hg38 graphs measured by mean absolute percentage error in VNTR length. Accuracy is averaged across 16 unrelated genomes.
rpgg.acc:	Genotyping accuracy using repeat-pangenome graphs measured by mean absolute percentage error in VNTR length. Accuracy is averaged across 16 unrelated genomes.
qual:	Averaged genotyping quality of the locus measured by R-squared of the linear fit between assembly and read k-mer counts. Quality score is averaged across 19 genomes each using its own repeat-genome graphs.
len.vst:	Vst statistic for VNTR length across the 5 populations
len.p:	ANOVA P-value of all populations having a same mean in VNTR length
mikmer.r2:	The variance explained in VNTR length by the most informative kmer
mikmer.ca:	The most informative canonical kmer wrt VNTR length
mikmer.ca’:	Reverse-complement of the most informative canonical kmer wrt VNTR length
eqtl:	Is an eVNTR
eqtl.n:	Number of tissues with this eVNTR
eqtl.info:	Association results for each discovery. FORMAT: GENE1:BETA1:QVAL1,GENE2:BETA2;QVAL2[,...](TISSUE1)[;...]
