# Supplementary material for: Profiling variable-number tandem repeat variation across populations using repeat-pangenome graphs
Source: Nat Commun. 2021 Jul 12;12:4250. doi: 10.1038/s41467-021-24378-0 (PMC8275641; doi:10.1038/s41467-021-24378-0)
Supplement: Supplementary file 13 — Reporting summary [file 41467_2021_24378_MOESM13_ESM.pdf]

nature research

Corresponding author(s): Mark Chaisson

Last updated by author(s): 2021-06-08

Reporting Summary

Nature Research wishes to improve the reproducibility of the work that we publish. This form provides structure for consistency and transparency in reporting. For further information on Nature Research policies, see our Editorial Policies and the Editorial Policy Checklist.

Statistics

For all statistical analyses, confirm that the following items are present in the figure legend, table legend, main text, or Methods section.

n/a	Confirmed	
☐	☒	The exact sample size (*n*) for each experimental group/condition, given as a discrete number and unit of measurement
☒	☐	A statement on whether measurements were taken from distinct samples or whether the same sample was measured repeatedly
☐	☒	The statistical test(s) used AND whether they are one- or two-sided<br>*Only common tests should be described solely by name; describe more complex techniques in the Methods section.*
☐	☒	A description of all covariates tested
☐	☒	A description of any assumptions or corrections, such as tests of normality and adjustment for multiple comparisons
☐	☒	A full description of the statistical parameters including central tendency (e.g. means) or other basic estimates (e.g. regression coefficient) AND variation (e.g. standard deviation) or associated estimates of uncertainty (e.g. confidence intervals)
☐	☒	For null hypothesis testing, the test statistic (e.g. *F*, *t*, *r*) with confidence intervals, effect sizes, degrees of freedom and *P* value noted<br>*Give P values as exact values whenever suitable.*
☒	☐	For Bayesian analysis, information on the choice of priors and Markov chain Monte Carlo settings
☒	☐	For hierarchical and complex designs, identification of the appropriate level for tests and full reporting of outcomes
☐	☒	Estimates of effect sizes (e.g. Cohen's *d*, Pearson's *r*), indicating how they were calculated

Our web collection on statistics for biologists contains articles on many of the points above.

Software and code

Policy information about availability of computer code

Data collection	Data were downloaded from public repositories using wget and ascp.
Data analysis	Our primary analysis was generated by danbing-tk, a c software program available on github (https://github.com/ChaissonLab/danbing-tk). Secondary analysis was conducted using Python3 scripts and snakemake (5.11.2), samtools (1.10), bedtools (2.29.2), minimap2 (2.17), scikit-learn (0.23.1), statsmodels (0.12.1), and pysam (0.15.3).

For manuscripts utilizing custom algorithms or software that are central to the research but not yet described in published literature, software must be made available to editors and reviewers. We strongly encourage code deposition in a community repository (e.g. GitHub). See the Nature Research guidelines for submitting code & software for further information.

Data

Policy information about availability of data

All manuscripts must include a data availability statement. This statement should provide the following information, where applicable:

- Accession codes, unique identifiers, or web links for publicly available datasets
- A list of figures that have associated raw data
- A description of any restrictions on data availability

All sequence read data are from public repositories. The GTEx data requires dbGaP access. Accession codes and web links are provided in the supplementary material. De novo assemblies, the corresponding repeat-pangenome, and the kmer dosage tables are available at https://doi.org/10.5281/zenodo.4758205.

Field-specific reporting

Please select the one below that is the best fit for your research. If you are not sure, read the appropriate sections before making your selection.

[x] Life sciences [ ] Behavioural & social sciences [ ] Ecological, evolutionary & environmental sciences

For a reference copy of the document with all sections, see nature.com/documents/nr-reporting-summary-flat.pdf

Life sciences study design

All studies must disclose on these points even when the disclosure is negative.

Sample size	Sample sizes were determined using all available data.
Data exclusions	No data were excluded.
Replication	We have successfully replicated our read mapping and Vst analysis using a set of individuals related to the 2,504 individuals sequenced by the 1000-genomes project. The eQTL experiments relied on the unique GTEx dataset, and were not replicated.
Randomization	This study did not an experimental effect of a treatment or condition, and so no randomization was performed.
Blinding	This study used a novel computational approach to discover properties of a data set, and blinding was not applicable.

Reporting for specific materials, systems and methods

We require information from authors about some types of materials, experimental systems and methods used in many studies. Here, indicate whether each material, system or method listed is relevant to your study. If you are not sure if a list item applies to your research, read the appropriate section before selecting a response.

Materials & experimental systems

n/a	Involved in the study	
[x]	[ ]	Antibodies
[ ]	[x]	Eukaryotic cell lines
[x]	[ ]	Palaeontology and archaeology
[x]	[ ]	Animals and other organisms
[x]	[ ]	Human research participants
[x]	[ ]	Clinical data
[x]	[ ]	Dual use research of concern

Methods

n/a	Involved in the study	
[x]	[ ]	ChIP-seq
[x]	[ ]	Flow cytometry
[x]	[ ]	MRI-based neuroimaging

Eukaryotic cell lines

Policy information about cell lines

Cell line source(s)	Coriell.
Authentication	We used sequencing data derived from cell lines, and did not authenticate cell lines.
Mycoplasma contamination	We used sequencing data derived from cell lines, and did not test for mycoplasma contamination. Sequence data were tested for outlier using principal component analysis.
Commonly misidentified lines (See ICLAC register)	*Name any commonly misidentified cell lines used in the study and provide a rationale for their use.*
